# Cytotoxic Polyketides Isolated from the Deep-Sea-Derived Fungus *Penicillium chrysogenum* MCCC 3A00292

**DOI:** 10.3390/md17120686

**Published:** 2019-12-05

**Authors:** Siwen Niu, Manli Xia, Mingliang Chen, Xiupian Liu, Zengpeng Li, Yunchang Xie, Zongze Shao, Gaiyun Zhang

**Affiliations:** 1 Key Laboratory of Marine Genetic Resources, State Key Laboratory Breeding Base of Marine Genetic Resources, Fujian Key Laboratory of Marine Genetic Resources, Third Institute of Oceanography, Ministry of Natural Resources, 184 Daxue Road, Xiamen 361005, China; niusi123@126.com (S.N.); xiaml0806@163.com (M.X.); liuxiupian@tio.org.cn (X.L.); lizengpeng@tio.org.cn (Z.L.); shaozongze@tio.org.cn (Z.S.); 2 College of life science, Jiangxi Normal University, Nanchang 330022, China; xieyunchang@jxnu.edu.cn

**Keywords:** Deep-sea-derived fungus, *Penicillium chrysogenum*, polyketides, versiol, cytotoxic activities

## Abstract

The chemical examination of the solid cultures of the deep-sea-derived fungus *Penicillium chrysogenum* MCCC 3A00292 resulted in the isolation of three new versiol-type analogues, namely peniciversiols A–C (**1**–**3**), and two novel lactone derivatives, namely penicilactones A and B (**6** and **7**), along with 11 known polyketides. The planar structures of the new compounds were determined by the comprehensive analyses of the high-resolution electrospray ionization mass spectroscopy (HRESIMS) and nuclear magnetic resonance (NMR) data, while their absolute configurations were resolved on the basis of comparisons of the experimental electronic circular dichroism (ECD) spectra with the calculated ECD data. Compound **1** is the second example of versiols featuring a 2,3-dihydropyran-4-one ring. Additionally, compounds **6** and **7** are the first representatives of *γ*-lactone derivatives constructed by a 1,3-dihydroxy-5-methylbenzene unit esterifying with the *α*-methyl-*γ*-hydroxy-*γ*-acetic acid *α*,*β*-unsaturated-*γ*-lactone moiety and *α*-hydroxy-*γ*-methyl-*γ*-acetic acid *α*,*β*-unsaturated-*γ*-lactone unit, respectively. All of the isolated compounds were evaluated for their cytotoxic activities against five human cancer cell lines of BIU-87, ECA109, BEL-7402, PANC-1, and Hela-S3. Compound **1** exhibited a selective inhibitory effect against the BIU-87 cell line (IC_50_ = 10.21 *μ*M), while compounds **4**, **5**, **8**, and **12**–**16** showed inhibitory activities against the ECA109, BIU-87, and BEL-7402 cell lines with the IC_50_ values ranging from 7.70 to > 20 *μ*M.

## 1. Introduction

Filamentous fungi are well known for their ability to produce structurally diverse secondary metabolites that feature interesting biological and pharmacological activities [1]. Recently, marine-derived-fungi, particularly deep-sea-derived fungi, have been recognized as a new source for a wide array of structurally fascinating secondary metabolites, and some of them have pharmaceutical activities [2,3,4]. For example, variecolortins A−C, three pairs of spirocyclic diketopiperazine enantiomers that were obtained from marine-derived fungus *Eurotium* sp. SCSIO F452, showed antioxidative and cytotoxic activities [5]. Simpterpenoid A, which is an unprecedented meroterpenoid that was obtained from mangrove-derived *Penicillium simplicissimum* MA-332, showed potent inhibitory activity against influenza neuraminidase in nanomolar quantities (IC_50_ = 8.1 nM) [6]. Taichunamide H, a new indole alkaloid, was isolated from mangrove-derived fungus *Aspergillus versicolor* HDN11-84 [7].

**Figure 1:**
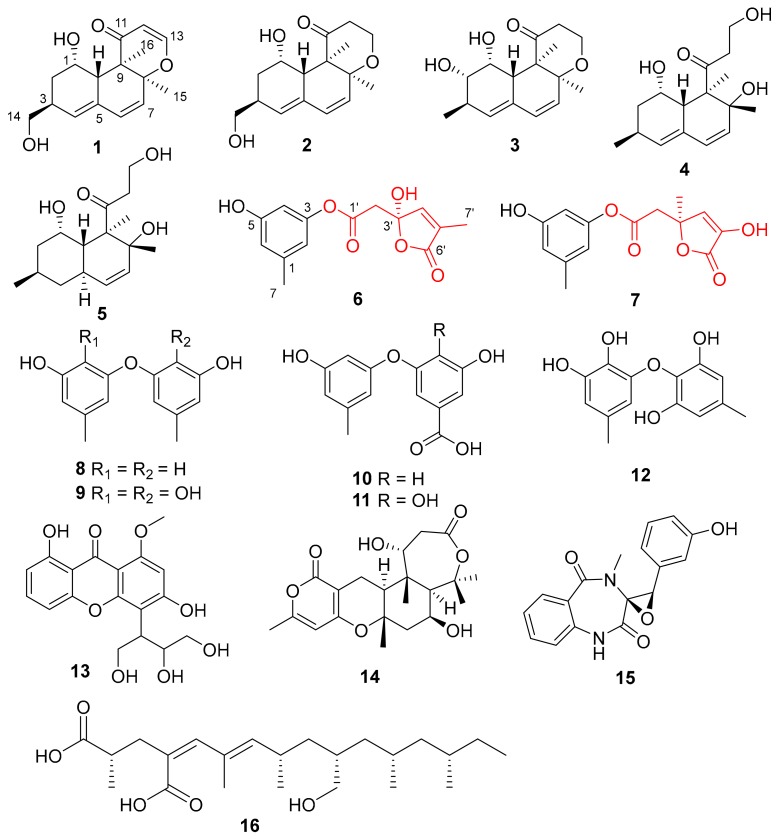
Chemical structures of **1**–**16** isolated from *P. chrysogenum* MCCC 3A00292.

Naturally occurring versiol derivatives are a rare class of fungal polyketides with an alkylated decalin nucleus. Structurally, versiol derivatives are classified into two types, according to the presence of a tetrahydropyran ring or not. As versiol was originally discovered from the fungus *Aspergillus versicolor* in 1975 [8], a total of 63 congeners have been discovered from several fungal genera [8,9,10,11,12,13,14,15,16,17,18,19,20,21,22]. Some of them showed interesting bioactivities, such as induced CD3^+^ T cell proliferation [11], antibacterial activities [12], the inhibition of colon cancer and melanoma cells [13], aromatase inhibitory activity [17], and induction neurite outgrowth in rat PC-12 cells [21]. As part of our continuing efforts to discover new and/or bioactivity secondary metabolites from the deep-sea-derived fungi [23,24,25,26,27,28], the fungus *Penicillium chrysogenum* MCCC 3A00292, isolated from South Atlantic Ocean at the depth of 2076 meters, was chosen for a systematic chemical investigation due to its rich metabolite profile in preliminary thin-layer chromatography (TLC) and high performance liquid chromatography (HPLC) screening. Chromatographic separation of the EtOAc extract of the fermented broth resulted in the isolation of 16 polyketides (**1**−**16**) (Figure 1), including three new versiol-type derivatives (peniciversiols A−C, **1**−**3**), and two novel *γ*-lactones (penicilactones A and B, **6** and **7**). In this study, the isolation, structure elucidation, and cytotoxic activities of these compounds are presented.

## 2. Results and Discussion

**Figure 2:**
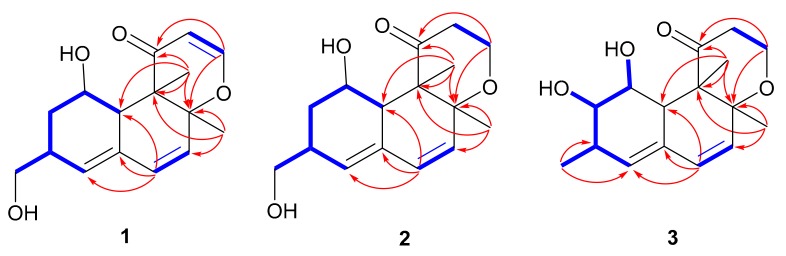
^1^H^-1^H COSY (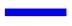) and key HMBC (
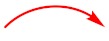
) correlations of compounds **1**−**3**.

Compound **1** was obtained as yellow orange oil, and its molecular formula was established to be C_16_H_20_O_4_ on the basis of the positive HRESIMS spectrum (*m/z* 299.1263, [M + Na]^+^) (Figure S1-1), requiring seven degrees of unsaturation. The ^1^H NMR spectrum exhibited two singlet methyls (*δ*_H_ 1.17 and 1.43), one hydroxymethyl (*δ*_H_ 3.47, 3.55), five olefinic protons (*δ*_H_ 5.38, 5.54, 5.92, 6.34, and 7.31), and one oxygenated methine (*δ*_H_ 3.93) (Table 1). The ^13^C NMR spectrum exhibited 16 carbon resonance signals that were classified with the help of the heteronuclear single quantum coherence (HSQC) spectrum, into six olefinic carbons (*δ*_C_ 104.8, 126.2, 132.5, 133.4, 135. 4, and 162.6) for three double bonds, a ketone carbonyl (*δ*_C_ 200.2), two methyls (*δ*_C_ 13.4 and 19.5), two methylenes including one oxygenated (*δ*_C_ 35.1 and 66.9), three methines (*δ*_C_ 35.6, 42.5, and 67.3), and two nonprotonated sp^3^ carbons (*δ*_C_ 52.0 and 86.6) (Table 1). The remaining degrees of unsaturation revealed a tricyclic ring system in **1** since four of seven degrees of unsaturation were accounted by for one carbonyl and three double bonds. Comprehensive analyses of the one-dimensional (1D) (^1^H and ^13^C) and two-dimensional (2D) [HSQC, ^1^H-^1^H homonuclear chemical shift correlation spectroscopy (^1^H^-1^H COSY), and heteronuclear multiple bond correlation (HMBC)] NMR spectra of **1** determined its structure to be related to the recently reported 12,13-dedihydroversiol [9]. The only difference was found by the presence of an additional hydroxy at C-14 (*δ*_C_ 66.9), as evidenced by the COSY cross-peaks of H_2_-2 (*δ*_H_ 1.33, 1.89)/H-3 (*δ*_H_ 2.68)/H-4 (*δ*_H_ 5.92)/H-3/H_2_-14 (*δ*_H_ 3.47, 3.55), in addition to the HMBC correlations from the oxygenated methylene H_2_-14 to C-2 (*δ*_C_ 35.1), C-3 (*δ*_C_ 35.6), and C-4 (*δ*_C_ 133.4) (Figure 2). An additional COSY correlation between H-12 (*δ*_H_ 5.38)/H-13 (*δ*_H_ 7.31), as well as the HMBC cross-peaks from H-13 to C-8 (*δ*_C_ 86.6) and C-11 (*δ*_C_ 200.2), and from H_3_-16 (*δ*_H_ 1.17) to C-8/C-9 (*δ*_C_ 52.0)/C-10 (*δ*_C_ 42.5)/C-11 discerned the presence of the *α*,*β*-unsaturated ketone unit residing at C-11, C-12 (*δ*_C_ 104.8), and C-13 (*δ*_C_ 162.6).

**Figure 3:**
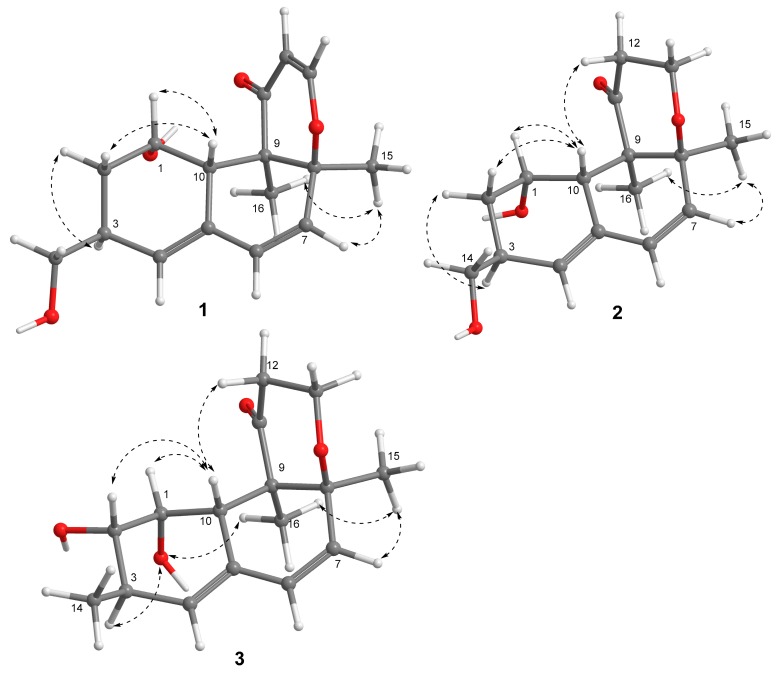
Selected NOESY correlations of compounds **1**−**3**.

**Figure 4:**
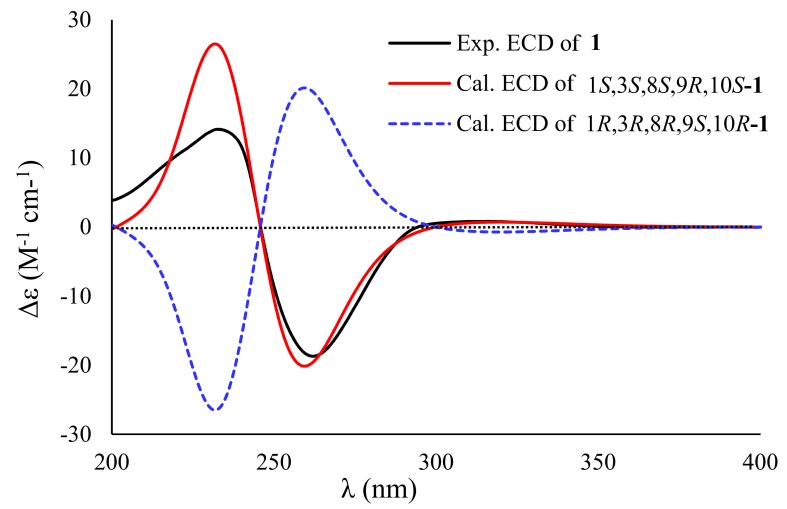
Experimental and calculated ECD spectra of **1** in MeOH.

The relative configuration of **1** was determined by the nuclear overhauser effect spectroscopy (NOESY) data and the coupling constants. The NOESY correlations from H-10 (*δ*_H_ 2.79) to H-1 (*δ*_H_ 3.93)/H-2a (*δ*_H_ 1.33) and from H-2b (*δ*_H_ 1.89) to H-3, in association with the small coupling constant *J*_H-1/H-10_ value (3.5 Hz) deduced the same orientation of H-1, H-10, and H_2_-14. Additional NOESY cross-peak of H_3_-15 (*δ*_H_ 1.43) and H_3_-16 and the absence of the NOESY relationship between H_3_-16 and H-10 revealed that CH_3_-15 and CH_3_-16 were in the opposite face toward H-10 (Figure 3). Therefore, the relative configuration of **1** was assigned as 1S*, 3S*, 8S*, 9R*, and 10S*. The theoretical ECD calculation was performed by the time-dependent density functional theory (TDDFT) method at the B3LYP/6-311 G(d,p) level while using the B3LYP/6-311 G(d,p)-optimized conformers after a conformational random search with the OPLS3 force field in order to establish its absolute configuration. The calculated ECD spectrum of 1*S*,3*S*,8*S*,9*R*,10*S*-**1** was in good agreement with the experimental ECD spectrum (Figure 4), indicating *S* configurations for C-1, C-3, C-8, and C-10, and *R* configuration for C-9. Thus, the structure of **1** was elucidated to be a 14-hydroxy-12,13-dedihydroversiol, which was given the trivial name peniciversiol A.

**Figure 5:**
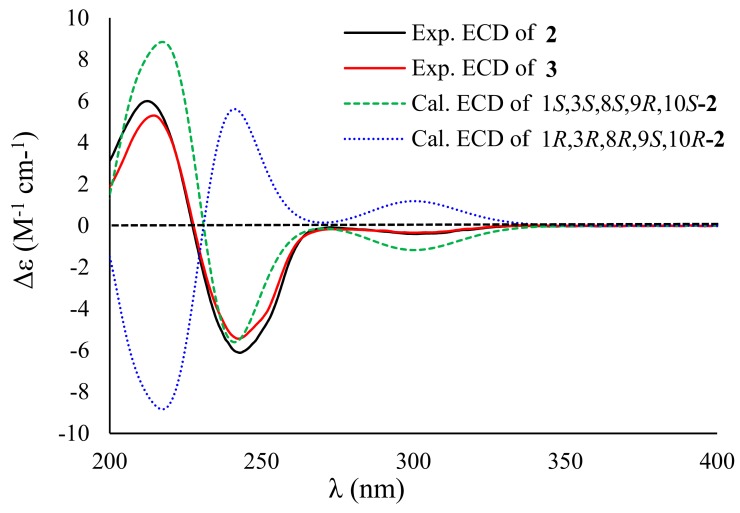
Experimental ECD spectra of **2** and **3** in MeOH and the calculated ECD spectra of 1*S*,3*S*,8*S*,9*R*,10*S*-**2** and 1*R*,3*R*,8*R*,9*S*,10*R*-**2**.

Compound **2** exhibited the molecular formula of C_16_H_22_O_4_, as established by the sodium adduct ion peak at *m/z* 301.1423 in the HRESIMS spectrum (Figure S2-1), requiring six degrees of unsaturation. The ^1^H NMR spectrum exhibited two singlet methyls (*δ*_H_ 1.08 and 1.22), four methylenes (two oxygenated at *δ*_H_ 3.47, 3.54, and 3.91, 4.06), three olefinic protons (*δ*_H_ 5.42, 5.84, and 6.21), and three methines (one oxygenated at *δ*_H_ 3.95), while the ^13^C NMR spectrum showed 16 carbon signals that involved two methyls (*δ*_C_ 13.6 and 20.9), four methylenes involving two oxygenated (*δ*_C_ 35.6, 39.9, 61.6, and 67.0), four olefinic carbons (*δ*_C_ 129.6, 131.4, 133.2, and 133.4), a ketone carbonyl (*δ*_C_ 213.7), three methines, including one oxygenated (*δ*_C_ 35.5, 43.1, and 67.2), and two nonprotonated sp^3^ carbons (*δ*_C_ 58.6 and 80.4). The above-mentioned NMR data were closely similar to those of **1**, with the exception of the presence of two additional methylenes in **2** instead of two olefinic carbons of **1**, revealing that **2** was a double bond hydrogenated derivative of **1**. The hydrogenated position at Δ^12,13^ was confirmed by the deshielding chemical shift of C-11 (Δ*δ*_C_ +13.5), the COSY correlation of H_2_-12 (*δ*_H_ 2.22, 3.03)/H_2_-13 (*δ*_H_ 3.91, 4.06), and the HMBC cross-peaks from H_2_-13 to C-8 (*δ*_C_ 80.4) and C-11 (*δ*_C_ 213.7) and from H_3_-16 (*δ*_H_ 1.08) to C-8/C-9 (*δ*_C_ 58.6)/C-10 (*δ*_C_ 43.1)/C-11 (Figure 2). The NOESY cross-peaks from H-10 (*δ*_H_ 3.44) to H-1 (*δ*_H_ 3.95)/H-2a (*δ*_H_ 1.44)/H-12b (*δ*_H_ 3.03), H-2b (*δ*_H_ 1.88) to H-3 (*δ*_H_ 2.67), and from H_3_-15 (*δ*_H_ 1.22) to H_3_-16, in association with the absence of a NOESY correlation between H_3_-16 and H-10 indicated that the relative configuration of **2** was same as that of **1** (Figure 3). The chiral centers of **2** were assigned to be identical to those of **1** by comparing the experimental and calculated ECD spectra of **2** (Figure 5). Therefore, **2** was established as a 12,13-hydrogenated derivative of **1** and named peniciversiol B.

The molecular formula of **3** was determined to be C_16_H_22_O_4_ on the basis of the positive HRESIMS spectrum (*m/z* 301.1420, [M + Na]^+^) and ^13^C NMR spectrum. The ^1^H and ^13^C NMR data closely resembled those of versiol [8,22], except for the presence of an extra oxymethine in **3** to replace a methylene of versiol, indicating that **3** was a hydroxylated derivative of versiol. The location of the hydroxy group at C-2 (*δ*_C_ 75.9) was discerned by the COSY relationships of 1-OH (*δ*_H_ 4.15)/H-1 (*δ*_H_ 3.52)/H-2 (*δ*_H_ 3.18)/2-OH (*δ*_H_ 4.54), in addition to the HMBC cross-peaks from H_3_-14 (*δ*_H_ 1.04) to the oxymethine C-2, C-3 (*δ*_C_ 33.0), and C-4 (*δ*_C_ 132.6) (Figure 2). The 2*α*-OH orientation was deduced by the NOESY cross-peak between H-10 (*δ*_H_ 3.38) and H-2. The relative configurations of the remaining stereogenic centers of **3** were identical to those of versiol on the basis of the NOESY correlations, as shown in Figure 3. On the basis of the nearly identical ECD spectra between **3** and **2**, the absolute configuration of **3** was assigned (Figure 5). Thus, the structure of **3** was elucidated as 2-hydroxylated derivative of versiol, which was named peniciversiol C.

**Figure 6:**
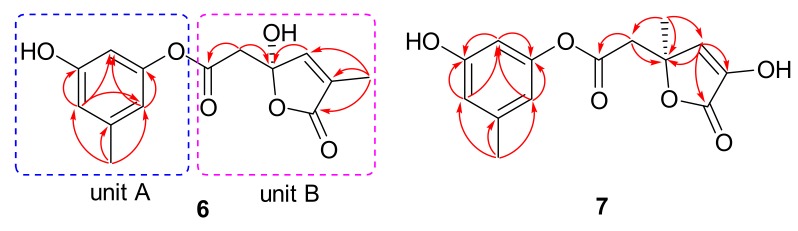
Selected HMBC correlations of **6** and **7**.

**Figure 7:**
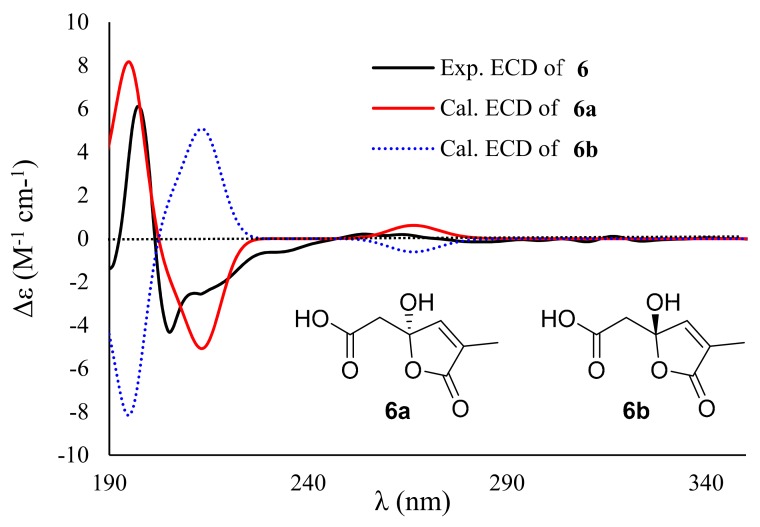
Experimental ECD spectra of **6** in CH_3_CN and the calculated ECD spectra of the corresponding simplified structures **6a** and **6b**.

Compound **6** exhibited a molecular formula of C_14_H_14_O_6_, as determined by the negative HRESIMS spectrum (*m/z* 277.0714, [M − H]^−^), requiring eight indices of hydrogen deficiency. The ^1^H NMR spectrum (Figure S4-2) showed three aromatic protons (*δ*_H_ 6.23, 6.25, and 6.38) for a 1,3,5-trisubstituted phenyl unit, two singlet methyls (*δ*_H_ 1.71 and 2.16), one olefinic proton (*δ*_H_ 7.33), and a methylene (*δ*_H_ 3.01, 3.15) (Table 2). The ^13^C NMR spectrum exhibited 14 carbon signals that were attributable to six aromatic carbons (*δ*_C_ 107.0, 113.1, 113.9, 139.4, 153.2, and 157.8) for a benzene ring, two olefinic carbons (*δ*_C_ 132.3 and 146.0) for a double bond, two carbonyl carbons (*δ*_C_ 169.2 and 170.5), two methyls (*δ*_C_ 10.0 and 21.1), a methylene (*δ*_C_ 41.9), and a nonprotonated sp^3^ carbon (*δ*_C_ 106.3) (Table 2). Because seven of eight degrees of unsaturation were accounted for by a benzene ring, a double bond, and two carbonyl groups, the remaining degree of unsaturation revealed the presence of the other cyclic ring in **6**. The COSY cross-peaks of H-2 (*δ*_H_ 6.25)/H-4 (*δ*_H_ 6.23)/H-6 (*δ*_H_ 6.38) and the HMBC correlations from H_3_-7 (*δ*_H_ 2.16) to C-1 (*δ*_C_ 139.4), C-2 (*δ*_C_ 113.9), and C-6 (*δ*_C_ 113.1), H-6 to C-2/C-4 (*δ*_C_ 107.0)/C-5 (*δ*_C_ 157.8), and from H-2 to C-3 (*δ*_C_ 153.2)/C-4/C-6 deduced a 3-oxygenated 5-methylphenol ring (unit A). Additional HMBC cross-peaks from H-2′ (*δ*_H_ 3.01, 3.15) to C-1′ (*δ*_C_ 169.2)/C-3′ (*δ*_C_ 106.3), H-4′ (*δ*_H_ 7.33) to C-3′, and from H_3_-7′ (*δ*_H_ 1.71) to C-4′ (*δ*_C_ 146.0), C-5′ (*δ*_C_ 132.3), and C-6′ (*δ*_C_ 170.5) determined the substructural segment of unit B (Figure 6). The connection of units A and B through an ester bond between C-3 and C-1′, as evidenced by the molecular formula of **6** and the shielding chemical shift of C-1′. The simplified structures of **6a** and **6b** were used for the ECD calculation because only one stereogenic center (C-3′) in **6** resided in unit B. The calculated ECD data of **6a** exhibited a nearly identical ECD curve to the experimental ECD spectrum of **6**, suggesting the 3′*S* configuration (Figure 7). Interestingly, compound **6**, featuring a rare *α*-methyl-*γ*-hydroxy-*γ*-acetic acid *α*,*β*-unsaturated-*γ*-lactone moiety fusing with 1,3-dihydroxy-5-methylbenzene unit by esterification, is a novel lactone derivative that is found in nature, which was named penicilactone A.

**Figure 8:**
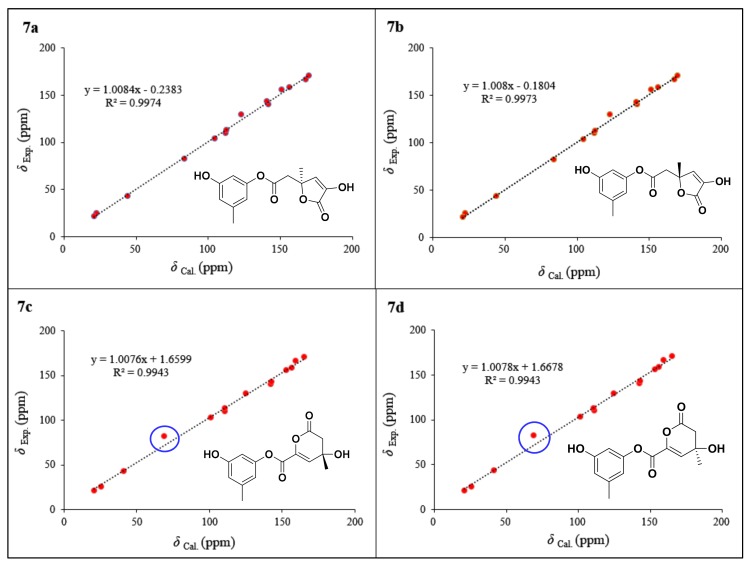
Correlation plots of experimental and calculated ^13^C NMR chemical shifts for the proposed structures of **7** (**7a**, **7b**, **7c**, and **7d**) at the mPW1PW91/6-311G + (2d,p) level.

**Figure 9:**
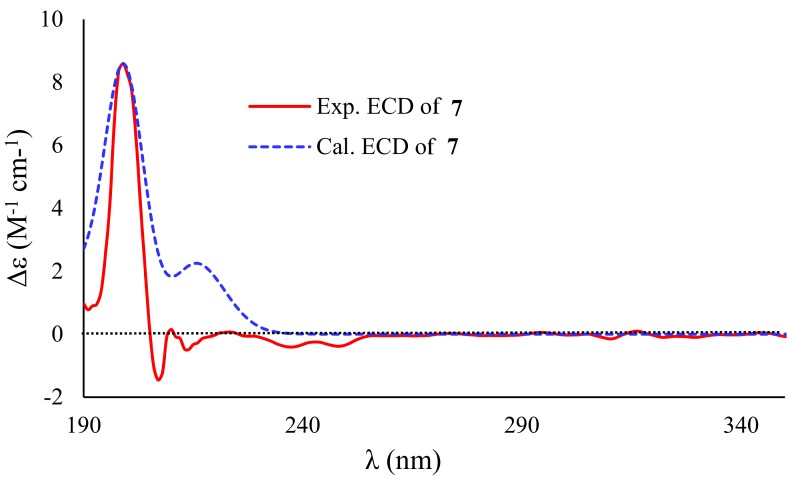
Experimental and calculated ECD spectra of **7** in CH_3_CN.

Compound **7** had the same molecular formula as that of **6**, as determined by the sodium adduct ion peak at *m/z* 301.0694 in the HRESIMS spectrum (Figure S5-1). The ^1^H and ^13^C NMR spectra of **7** showed the characteristic resonance signals for the 1,3,5-trisubstituted phenyl unit, which was the same to that of **6**. The obvious differences were found by the deshielding chemical shifts of C-5′ (Δ*δ*_C_ +10.6) and C-7′ (Δ*δ*_C_ +15.0), and the shielding chemical shifts of C-3′ (Δ*δ*_C_ −24.3), C-4′ (Δ*δ*_C_ −16.5), and C-6′ (Δ*δ*_C_ −4.2) compared with the corresponding ^13^C NMR data of **6**, revealing the position of the methyl at C-5′ and hydroxy at C-3′ in **6** were transposed in **7**. HMBCs confirmed the assumption from H_3_-7′ (*δ*_H_ 1.49) to C-2′ (*δ*_C_ 43.1), C-3′ (*δ*_C_ 82.0), and C-4′ (*δ*_C_ 129.5), H-4′ (*δ*_H_ 6.69) to C-3′/C-5′ (*δ*_C_ 142.9)/C-6′ (*δ*_C_ 166.3), and from H_2_-2′ (*δ*_H_ 2.74, 2.80) to C-1′ (*δ*_C_ 170.5) and C-3′ (Figure 6). The other possible structure might be constructed by an ester bond between C-5′ and C-1′ to form a *δ*-lactone ring because the D_2_O exchangeable proton 5′-OH was not detected in the ^1^H NMR spectrum of **7**, as shown in Figure 8 (**7c** or **7d**). When considering the deshielding chemical shifts of C-3′, the aforementioned candidate (**7c** or **7d**) was less likely than **7a** or **7b**. However, this assumption needed additional solid evidences. Accordingly, the ^13^C NMR chemical shifts of the four possible structures of **7** (**7a**−**7d**) were calculated by the GIAO method at the mPW1PW91/6-311G + (2d,p) level using the Gaussian 09 following the protocol adapted from Michael et al. [29]. The calculated ^13^C NMR chemical shifts of **7a** and **7b** were in good match with the experimental data, which excluded candidates **7c** and **7d** (Figure 8). The correlation coefficient (R_2_) of **7a** (0.9974) and **7b** (0.9973) were higher than those of **7c** (0.9943) and **7d** (0.9943), and the average absolute deviation of **7a** (2.66 ppm) and **7b** (2.68 ppm) were obviously lower than those of **7c** (4.49 ppm) and **7d** (4.51 ppm). Finally, the structure of **7a** was assigned for **7** on the basis of the experimental and calculated ECD spectra (Figure 9). Noteworthily, compound **7**, bearing an *α*-hydroxy-*γ*-methyl-*γ*-acetic acid *α*,*β*-unsaturated-*γ*-lactone moiety fusing with 1,3-dihydroxy-5-methylbenzene unit by esterification, is a novel lactone derivative that was discovered in nature, which was given the trivial name penicilactone B.

Additionally, eleven known compounds were determined to be decumbenone A (**4**) [22], decumbenone B (**5**) [22], 3,3′-dihydroxy-5,5′-dimethyldiphenyl ether (**8**) [30,31], aspermutarubrol (**9**) [32], 3-hydroxy-5-(3-hydroxy-5-methylphenoxy)benzoic acid (**10**) [33], 3,4-dihydroxy-5-(3-hydroxy-5-methylphenoxy)benzoic acid (**11**) [33], violaceol-Ⅱ (**12**) [34,35], 3,8-dihydroxy-4-(2,3-dihydroxy-1-hydroxymethylpropyl)-1-methoxyxanthone (**13**) [36], asperdemin (**14**) [37], cyclopenol (**15**) [38,39], and radiclonic acid (**16**) [40], on the basis of the comparisons of their NMR and specific rotation data with those that were reported in the literature.

As some of the versiol derivatives showed potent cytotoxic activities in the literature, all of the isolated compounds (**1**−**16**) were evaluated for their cytotoxic effects against five human cancer cell lines of BIU-87, ECA109, BEL-7402, PANC-1, and Hela-S3 by the MTT method at the initial concentration of 20 *μ*M. All of the compounds displayed the inhibition rates less than 50% against two cancer cell lines of PANC-1 and Hela-S3, while compounds **1**, **4**, **5**, **8**, and **12**−**16**, which exhibited inhibition rates more than 50%, were further tested to establish their IC_50_ values. Compound **1** showed significantly selective inhibitory effect against BIU-87 cell line with an IC_50_ value of 10.21 *μ*M, **12** and **16** selective inhibition ECA109 cell line with IC_50_ values of 8.95 and 7.70 *μ*M, respectively, and **15** selective inhibition BEL-7402 (IC_50_ = 7.81 *μ*M) and BIU-87 (IC_50_ = 8.34 *μ*M) cell lines (Table 3). A primary analyses of the structure-activity relationships revealed that the double bond at Δ^12,13^ in **1** is necessary for the inhibition of the BIU-87 cell line, as exampled that **1** has potent inhibitory activity, while **2** displayed no activity.

## 3. Materials and Methods

### 3.1. General Experimental Procedures

The specific rotations were recorded on a Rudolph Autopol VI automatic polarimeter (Rudolph Reaearch Analytical) in MeOH at 24 °C. The UV spectra were measured in MeOH on a UV8000 UV/Vis spectrophotometer (Shanghai Metash instrument, Shanghai, China). The ECD data were measured on a Chirascan Spectrometer (Applied Photophysics, Surrey, UK) in MeOH or MeCN. The ^1^H, APT, HSQC, COSY, HMBC, and NOESY spectra were detected by the Bruker AV-400 NMR spectrometers (Bruker, Fällanden, Switzerland). The chemical shifts are expressed in *δ* referenced to the solvent residual peaks of CD_3_OD (*δ*_H_ 3.31 and *δ*_C_ 49.0) and DMSO-*d*_6_ (*δ*_H_ 2.50 and *δ*_C_ 39.5). The Xevo G2 Q-TOF mass spectrometer (Waters, Milford, MA, USA) was used for recording the HRESIMS spectra. Column chromatography (CC) was carried out over silica gel (200−300 mesh, Qingdao Marine Chemistry Co., Ltd., Qingdao, China), ODS (50 *μ*m, YMC Co., Ltd., Kyoto, Japan), and Sephadex LH-20 (GE Healthcare, Pittsburgh, PA, USA), respectively. Precoated silica gel plates (Qingdao Marine Chemistry Co., Ltd., Qingdao, China) were used for TLC analyses. The solvents used for isolation were all of analytical grade.

### 3.2. Fungal Material

The fungus *Penicillium chrysogenum* MCCC 3A00292 was isolated from the deep-sea sediment of the South Atlantic Ocean (GPS 11.4293° W, 20.8914° S) at the depth of 2076 meters during the Comra 22nd oceanic cruise in May 2011. The fungal strain was identified as *P. chrysogenum* on the basis of the ITS region sequence, which has 100% similarity to that of *P. chrysogenum* A096. The ITS gene sequence of this fungus was deposited in the GenBank and given the accession no. MN481191. The voucher strain is preserved at the Marine Culture Collection of China (MCCC), Third Institute of Oceanography, Ministry of Natural Resources, China, and given the deposited number MCCC 3A00292.

### 3.3. Fermentation, Extraction, and Isolation

For large-scaled fermentation, the fresh mycelia was obtained from the PDA plates at 25 °C for three days and then inoculated to 2 × 500 mL Erlenmeyer flasks each containing 200 mL PDB medium. Subsequently, they were cultured in rotary shaker at 180 rpm and 28 °C for four days to obtain seed medium. Finally, the seed cultures were inoculated to 25 × 1 L Erlenmeyer flasks, each containing 80 g rice and 120 mL of seawater. After incubation for 30 days under static conditions at 25 °C, the fermented solid mash was mechanically fragmented and extracted with EtOAc three times to afford an extract (22 g).

The extract was subjected to CC over silica gel eluting with a gradient of petroleum ether (PE) and EtOAc (1:0 → 0:1) to yield six fractions (Fr.1−Fr.6). Fraction Fr.2 (325 mg) was further purified by CC over ODS eluting with MeOH/H_2_O (4:1→1:0) to yield eight subfractions (SFr.2-1−SFr.2-8). SFr.2-4 was further chromatographed by repeated silica gel CC eluting with CH_2_Cl_2_/MeOH (1:0→0:1), CH_2_Cl_2_/acetone (50:1→0:1), in association with recrystallization to obtain **2** (17.5 mg), **3** (7.2 mg), **11** (1.9 mg), and **15** (25.6 mg). Fraction Fr.3 (189 mg) was subjected to ODS CC with the mobile phase of MeOH in H_2_O (10:1→1:0) to yield four subfractions (SFr.3-1−SFr.3-4). Subfraction SFr.3-2 was separated by CC on Sephadex LH-20 (MeOH) to afford two subfractions. The former was further purified by a semipreparative HPLC with the mobile phase of CH_3_CN/H_2_O (23:77) to obtain **6** (3.2 mg) and **7** (1.8 mg), while the latter was further subjected to CC on silica gel (CH_2_Cl_2_/MeOH, 100:1→0:1) to yield **12** (25.5 mg) and **13** (14.6 mg). Fraction Fr.4 (222 mg) was subjected to CC over ODS with the mobile phase of MeOH/H_2_O (5%→100%) to afford three subfractions (SFr.4-1−SFr.4-3). SFr.4-1 was further purified by CC over Sephadex LH-20 eluting with MeOH and by silica gel CC eluting with CH_2_Cl_2_/MeOH (80:1→0:1) to yield **4** (12.2 mg) and **8** (42.5 mg). SFr.4-2 was further separated by repeated CC over silica gel eluting with PE/EtOAc (1:0→0:1), PE/acetone (100:1→0:1), and CH_2_Cl_2_/MeOH (40:1→0:1), respectively, to yield **9** (28.1 mg), **10** (12.3 mg), and **14** (4.2 mg). ODS CC using MeOH in H_2_O as the mobile phase (5%→100%) chromatographed Fr.5 (172 mg) to yield six subfractions (SFr.6-1−SFr.6-6) and **16** (48.2 mg). Subfraction SFr.6-4 was further separated by CC on Sephadex LH-20 (MeOH) and semipreparative HPLC eluting with MeOH/H_2_O (3:7→7:3) to yield **1** (4.0 mg) and **5** (9.2 mg).

Peniciversiol A (**1**): yellow orange oil; [*α*${]}_{D}^{24}$ −223 (*c* 0.12, MeOH); UV (MeOH) *λ*_max_ (log *ε*) 239 (4.02) nm; ECD (MeOH) *λ*_max_ (Δ*ε*) 234 (+14.15), 263 (−18.64) nm; ^1^H and ^13^C NMR data, see Table 1; HRESIMS *m/z* 299.1263 [M + Na]^+^ (calcd for C_16_H_20_O_4_Na, 299.1259), *m/z* 575.2617 [2M + Na]^+^ (calcd for C_32_H_40_O_8_Na, 575.2621).

Peniciversiol B (**2**): yellow orange oil; [*α*${]}_{D}^{24}$ −58 (*c* 0.54, MeOH); UV (MeOH) *λ*_max_ (log *ε*) 241 (3.98) nm; ECD (MeOH) *λ*_max_ (Δ*ε*) 213 (+5.98), 243 (−6.12) nm; ^1^H and ^13^C NMR data, see Table 1; HRESIMS *m/z* 301.1423 [M + Na]^+^ (calcd for C_16_H_22_O_4_Na, 301.1416).

Peniciversiol C (**3**): yellow orange oil; [*α*${]}_{D}^{24}$ −89 (*c* 0.25, MeOH); UV (MeOH) *λ*_max_ (log *ε*) 241 (3.98) nm; ECD (MeOH) *λ*_max_ (Δ*ε*) 215 (+5.29), 243 (−5.43) nm; ^1^H and ^13^C NMR data, see Table 1; HRESIMS *m/z* 301.1420 [M + Na]^+^ (calcd for C_16_H_22_O_4_Na, 301.1416), *m/z* 579.2927 [2M + Na]^+^ (calcd for C_32_H_44_O_8_Na, 579.2934).

Penicilactone A (**6**): colorless oil; [*α*${]}_{D}^{24}$ −3 (*c* 0.31, MeOH); UV (MeOH) *λ*_max_ (log *ε*) 213 (3.77), 274 (3.19) nm; ECD (CH_3_CN) *λ*_max_ (Δ*ε*) 197 (+6.09), 205 (−4.31) nm; ^1^H and ^13^C NMR data, see Table 2; HRESIMS *m/z* 277.0714 [M − H]^−^ (calcd for C_14_H_13_O_6_, 277.0712).

Penicilactone B (**7**): colorless oil; [*α*${]}_{D}^{24}$ −1 (*c* 0.07, MeOH); UV (MeOH) *λ*_max_ (log *ε*) 218 (3.71), 273 (3.13) nm; ECD (CH_3_CN) *λ*_max_ (Δ*ε*) 199 (+8.59), 207 (−1.46) nm; ^1^H and ^13^C NMR data, see Table 2; HRESIMS *m/z* 301.0694 [M + Na]^+^ (calcd for C_14_H_14_O_6_Na, 301.0688).

### 3.4. Cytotoxicity Assay

All of the isolated compounds were evaluated for their cytotoxic activities against five cancer cell lines (BIU-87, ECA109, BEL-7402, PANC-1, and Hela-S3) while using the MTT method. In brief, the BIU-87, ECA109, and BEL-7402 cells were cultured in RPMI 1640 medium, and the PANC-1 and Hela-S3 cells were cultured in DMEM medium, which was supplemented with 10% FBS (fetal bovine serum) in a humidified incubator (5% CO_2_ at 37 °C). The cells were seeded in 96-well plates at a density of 5000 cells/well. After 24 h incubation, the tested compounds were added, and incubation continued for another 48 h. Subsequently, 10 *μ*L of 3-(4,5-dimethylthiazol-2-yl)-2,5-diphenyltetrazolium bromide (MTT) (5 mg/mL) was added into the medium and then incubated for another 1 h. Afterwards, the medium was removed and 150 *μ*L of DMSO was added. The OD absorbance values of each well were recorded at 490 nm while using a SpectraMax M5 microplate reader (Molecular Devices). The isolated compounds were tested at five concentrations (0, 2.5, 5, 10, and 20 *μ*M) with a final DMSO concentration of 0.5% (v/v) in each well. Dose-response curves were plotted to determine the IC_50_ values that were based on the averaged values of three parallel experiments while using the GraphPad Prism 7.0 software (San Diego, CA, USA).

### 3.5. Computation Section

Systematically conformational searches were performed by the Maestro 10.2 program (New York, NY, USA) at the OPLS3 molecular mechanics force field within an energy window of 3.0 kcal/mol. The conformers were further optimized by the B3LYP/6-311G (d,p) level while using Gaussian 09 software (Wallingford, CT, USA) [41]. The conformers with the Boltzmann population of over 1% were chosen for the ECD calculations. The theoretical calculations of the ECD data were conducted while using the TDDFT method at the B3LYP/6-311G (d,p) level with CPCM model in MeOH for **1** and **2** and in CH_3_CN for **6** and **7**. The ECD spectra were simulated using the SpecDis [42] by applying the Gaussian band shapes with sigma = 0.3 eV, and the finally calculated ECD data were weighted and then summed up of each stable conformers according to the Boltzmann population. The ^13^C NMR chemical shifts of compounds **7a**–**7d** were calculated with the GIAO method at the mPW1PW91/6-311G + (2d,p) levels by the Gaussian 09 following the protocol adapted from Michael et al [29]. Finally, the calculated ^13^C NMR chemical shift values were averaged according to the Boltzmann distribution for each conformer and then fitted to the experimental values by linear regression.

## 4. Conclusions

In the present work, three new versiol-type congeners peniciversiols A−C (**1**−**3**) and two novel *γ*-lactone derivatives penicilactones A and B (**6** and **7**) were isolated from the solid cultures of the deep-sea-derived fungus *Penicillium chrysogenum* MCCC 3A00292, along with 11 known polyketides The comprehensive analyses of the HRESIMS and NMR data, in association with comparisons of the experimental and calculated ECD spectra for configurational assignments, determined the structures of new compounds. Compound **1** represents the second example of versiols featuring a 2,3-dihydropyran-4-one ring, while **6** and **7** are first representatives of the *γ*-lactone derivatives constructed by a 1,3-dihydroxy-5-methylbenzene unit separately esterifying with the *α*-methyl-*γ*-hydroxy-*γ*-acetic acid *α*,*β*-unsaturated-*γ*-lactone moiety and *α*-hydroxy-*γ*-methyl-*γ*-acetic acid *α*,*β*-unsaturated-*γ*-lactone unit. Compound **1** exhibited a selective inhibitory effect against the BIU-87 cell line (IC_50_ = 10.21 *μ*M), while **16** showed potent inhibitory activities against the ECA109, BIU-87, and BEL-7402 cell lines with the IC_50_ values of 7.70, 12.47, and 13.75 *μ*M, respectively, and compounds **4**, **5**, **8**, and **12**−**15** showed different inhibitory activities against the BIU-87, ECA109, and BEL-7402 cell lines, with the IC_50_ values ranging from 7.81 to > 20 *μ*M, indicating their potential applications for further development as antitumor lead compounds.

## Figures and Tables

**Table 1:** ^1^H (400 MHz) and ^13^C (100 MHz) NMR data of **1**−**3** (*δ* in ppm, *J* in Hz within parentheses).

no.	1 ^a^		2 ^a^		3 ^b^	
	*δ*_H_	*δ*_C_	*δ*_H_	*δ*_C_	*δ*_H_	*δ*_C_
1	3.93, t (3.5)	67.3, CH	3.95, t (3.2)	67.2, CH	3.52, q (2.4)	70.1, CH
2	1.89, dtd (12.5, 4.8, 1.3); 1.33, td (12.5, 1.6)	35.1, CH_2_	1.88, dtd (12.6, 5.0, 1.3); 1.44, td (12.6, 1.8)	35.6, CH_2_	3.18, ddd (8.6, 5.9, 1.7)	75.9, CH
3	2.68, m	35.6, CH	2.67, m	35.5, CH	2.36, m	33.0, CH
4	5.92, br s	133.4, CH	5.84, br s	131.4, CH	5.50, t (2.3)	132.6, CH
5		132.5, C		133.4, C		130.2, C
6	6.34, d (9.6)	135.4, CH	6.21, d (9.6)	133.2, CH	6.15, d (9.6)	130.7, CH
7	5.54, d (9.6)	126.2, CH	5.42, d (9.6)	129.6, CH	5.39, d (9.6)	129.0, CH
8		86.6, C		80.4, C		78.1, C
9		52.0, C		58.6, C		56.5, C
10	2.79, m	42.5, CH	3.44, t (3.2)	43.1, CH	3.38, overlap	41.1, CH
11		200.2, C		213.7, C		210.2, C
12	5.38, d (5.9)	104.8, CH	3.03, ddd (14.6, 12.2, 8.8); 2.22, dd (14.6, 3.0)	39.9, CH_2_	2.98, ddd (14.2, 12.0, 8.9); 2.11, dd (14.2, 3.0)	38.4, CH_2_
13	7.31, d (5.9)	162.6, CH	4.06, dd (11.9, 8.3); 3.91, dd (11.9, 3.5)	61.6, CH_2_	3.98, dd (11.8, 8.8); 3.79, td (11.8, 3.2)	60.0, CH_2_
14	3.55, dd (10.6, 5.9); 3.47, dd (10.6, 6.5)	66.9, CH_2_	3.54, dd (10.5, 6.2); 3.47, dd (10.5, 6.6)	67.0, CH_2_	1.04, d (7.2)	17.8, CH_3_
15	1.43, s	19.5, CH_3_	1.22, s	20.9, CH_3_	1.11, s	20.1, CH_3_
16	1.17, s	13.4, CH_3_	1.08, s	13.6, CH_3_	0.99, s	13.2, CH_3_
1-OH					4.15, d (3.3)	
2-OH					4.54, d (5.9)	

^a^ Recorded in CD_3_OD. ^b^ Recorded in DMSO-*d*_6_.

**Table 2:** ^1^H (400 MHz) and ^13^C (100 MHz) NMR spectroscopic data of **6** and **7** in DMSO-*d*_6_.

Position	6		7	
	*δ*_H_, Mult (*J* in Hz)	*δ*_C_, Type	*δ_H_,* Mult (*J* in Hz)	*δ*_C_, Type
1		139.4, C		140.4, C
2	6.25, br s	113.9, CH	6.45, br s	112.7, CH
3		153.2, C		155.7, C
4	6.23, br s	107.0, CH	6.38, br s	103.0, CH
5		157.8, C		158.5, C
6	6.38, br s	113.1, CH	6.45, br s	109.7, CH
7	2.16, s	21.1, CH_3_	2.22, s	21.1, CH_3_
1′		169.2, C		170.5, C
2′	3.15, d (15.5); 3.01, d (15.5)	41.9, CH_2_	2.80, d (15.5); 2.74, d (15.5)	43.1, CH_2_
3′		106.3, C		82.0, C
4′	7.33, br s	146.0, CH	6.69, s	129.5, CH
5′		132.3, C		142.9, C
6′		170.5, C		166.3, C
7′	1.71, s	10.0, CH_3_	1.49, s	25.0, CH_3_

**Table 3:** Cytotoxic activities of compounds **1, 4, 5, 8,** and **12**−**16** against five cancer cell lines.

	IC_50_ (*μ*M)				
Compounds	BIU-87	ECA109	BEL-7402	PANC-1	Hela-S3
**1**	10.21	>20	>20	>20	>20
**4**	>20	12.41	>20	>20	>20
**5**	>20	15.60	>20	>20	>20
**8**	16.41	>20	>20	>20	>20
**12**	>20	8.95	>20	>20	>20
**13**	>20	>20	15.94	>20	>20
**14**	>20	>20	12.75	>20	>20
**15**	8.34	>20	7.81	>20	>20
**16**	12.47	7.70	13.75	>20	>20

## Supplementary Materials

The following are available online at https://www.mdpi.com/1660-3397/17/12/686/s1. HRESIMS, ^1^H, APT, HSQC, COSY, and HMBC spectra of **1**−**3**, **6**, and **7**; NOESY spectra of **1**−**3**.
