# Supplementary material for: Cytotoxic Polyketides Isolated from the Deep-Sea-Derived Fungus Penicillium chrysogenum MCCC 3A00292
Source: Mar Drugs. 2019 Dec 5;17(12):686. doi: 10.3390/md17120686 (PMC6950755; doi:10.3390/md17120686)
Supplement: Supplementary file 1 [file marinedrugs-17-00686-s001.pdf]

Supporting Information

Cytotoxic Polyketides Isolated from the Deep-Sea-Derived Fungus *Penicillium chrysogenum* MCCC 3A00292

Siwen Niu [1,†], Manli Xia [1,†], Mingliang Chen [1,*], Xiupian Liu [1], Zengpeng Li [1], Yunchang Xie [2], Zongze Shao [1] and Gaiyun Zhang [1,*]

1 Key Laboratory of Marine Genetic Resources, State Key Laboratory Breeding Base of Marine Genetic Resources, Fujian Key Laboratory of Marine Genetic Resources, Third Institute of Oceanography, Ministry of Natural Resources, 184 Daxue Road, Xiamen 361005, China; niusi123@126.com (S.N.); xiaml0806@163.com (M.X.); liuxiupian@tio.org.cn (X.L.); lizengpeng@tio.org.cn (Z.L.); shaozongze@tio.org.cn (Z.S.)

2 College of life science, Jiangxi Normal University, Nanchang 330022, China; xieyunchang@jxnu.edu.cn

* Correspondence: mlchen_gg@tio.org.cn (M.C.); zhgyun@tio.org.cn (G.Z.); Tel.: +86-592-2195393 (M.C.); +86-592-2195833 (G.Z.)

† The authors contributed equally to this work.

Contents

Figure S1-1. ^{1}H NMR spectrum of **1** in CD_3OD (400 MHz).

Figure S1-2. ^{13}C NMR spectrum of **1** in CD_3OD (100 MHz).

Figure S1-3. HSQC spectrum of **1** in CD_3OD.

Figure S1-4. COSY spectrum of **1** in CD_3OD.

Figure S1-5. HMBC spectrum of **1** in CD_3OD.

Figure S1-6. NOESY spectrum of **1** in CD_3OD.

Figure S2-1. ^{1}H NMR spectrum of **2** in CD_3OD (400 MHz).

Figure S2-2. ^{13}C NMR spectrum of **2** in CD_3OD (100 MHz).

Figure S2-3. HSQC spectrum of **2** in CD_3OD.

Figure S2-4. COSY spectrum of **2** in CD_3OD.

Figure S2-5. HMBC spectrum of **2** in CD_3OD.

Figure S2-6. NOESY spectrum of **2** in CD_3OD.

Figure S3-1. ^{1}H NMR spectrum of **3** in DMSO-d_6 (400 MHz).

Figure S3-2. ^{13}C NMR spectrum of **3** in DMSO-d_6 (100 MHz).

Figure S3-3. HSQC spectrum of **3** in DMSO-d_6.

Figure S3-4. COSY spectrum of **3** in DMSO-d_6.

Figure S3-5. HMBC spectrum of **3** in DMSO-d_6.

Figure S3-6. NOESY spectrum of **3** in DMSO-d_6.

Figure S4-1. ^{1}H NMR spectrum of **6** in DMSO-d_6 (400 MHz).

Figure S4-2. ^{13}C NMR spectrum of **6** in DMSO-d_6 (100 MHz).

Figure S4-3. HSQC spectrum of **6** in DMSO-d_6.

Figure S4-4. COSY spectrum of **6** in DMSO-d_6.

Figure S4-5. HMBC spectrum of **6** in DMSO-d_6.

Figure S5-1. ^{1}H NMR spectrum of **7** in DMSO-d_6 (400 MHz).

Figure S5-2. ^{13}C NMR spectrum of **7** in DMSO-d_6 (100 MHz).

Figure S5-3. HSQC spectrum of **7** in DMSO-d_6.

Figure S5-4. COSY spectrum of **7** in DMSO-d_6.

Figure S5-5. HMBC spectrum of **7** in DMSO-d_6.

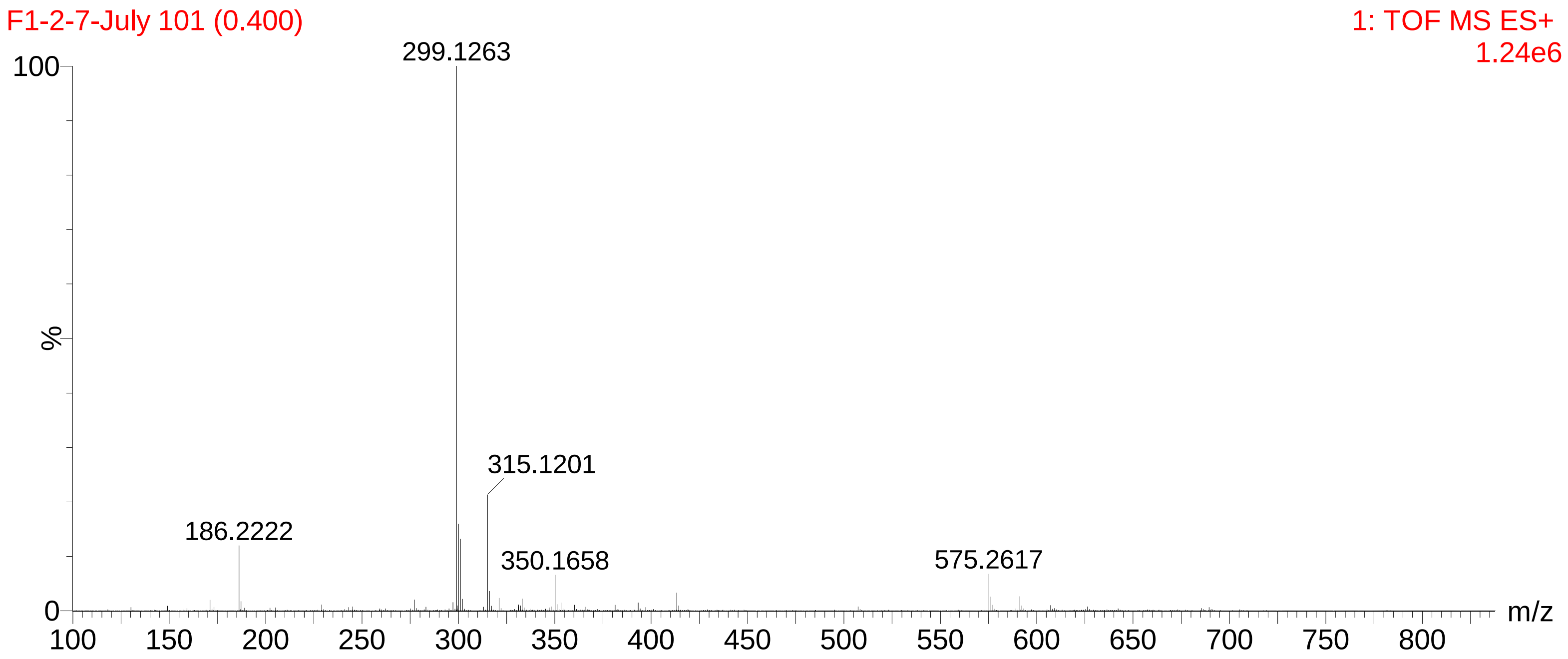

Figure S1-1. HRESIMS spectrum of **1**.

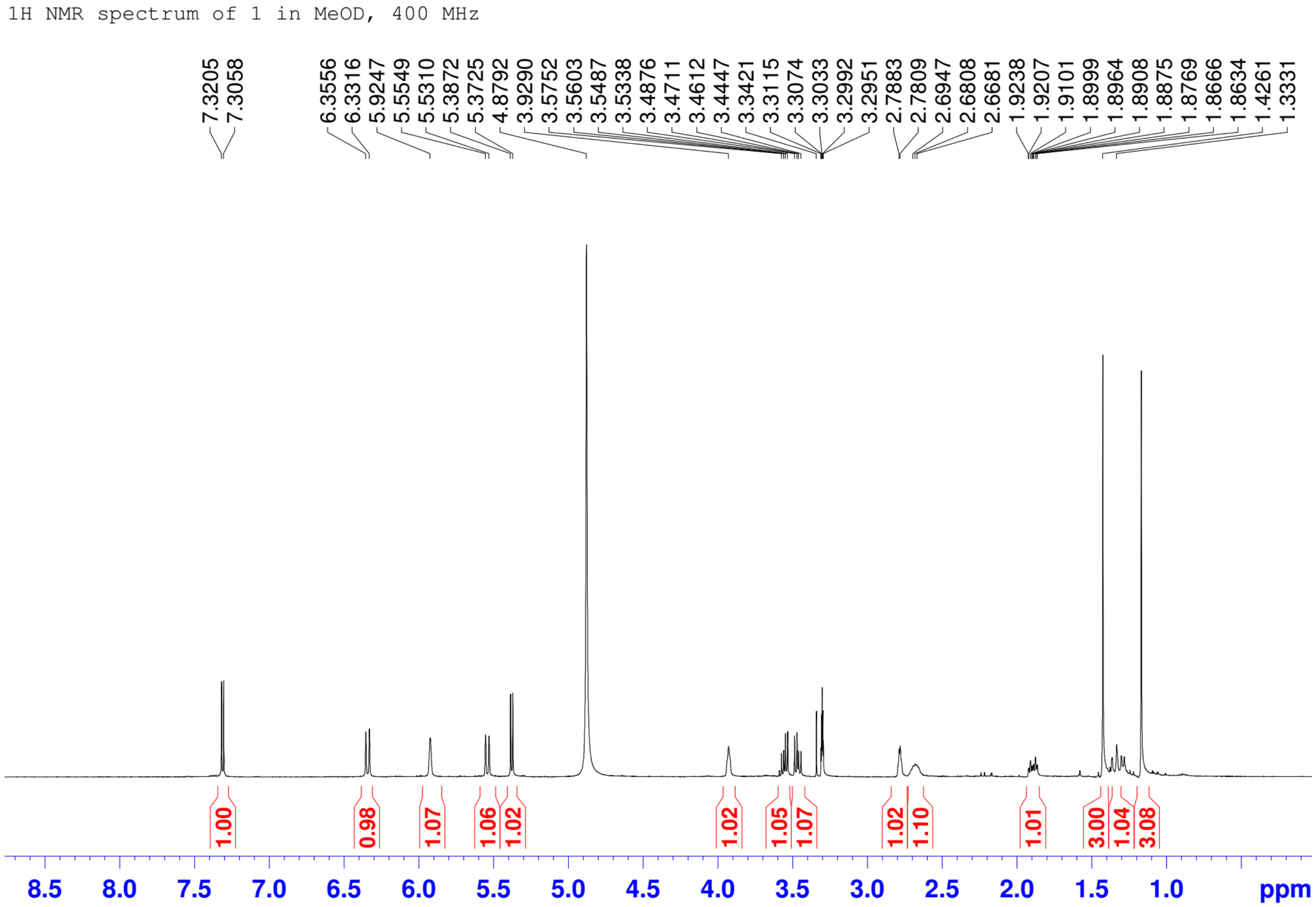

Figure S1-2. ^{1}H NMR spectrum of **1** in CD_3OD (400 MHz).

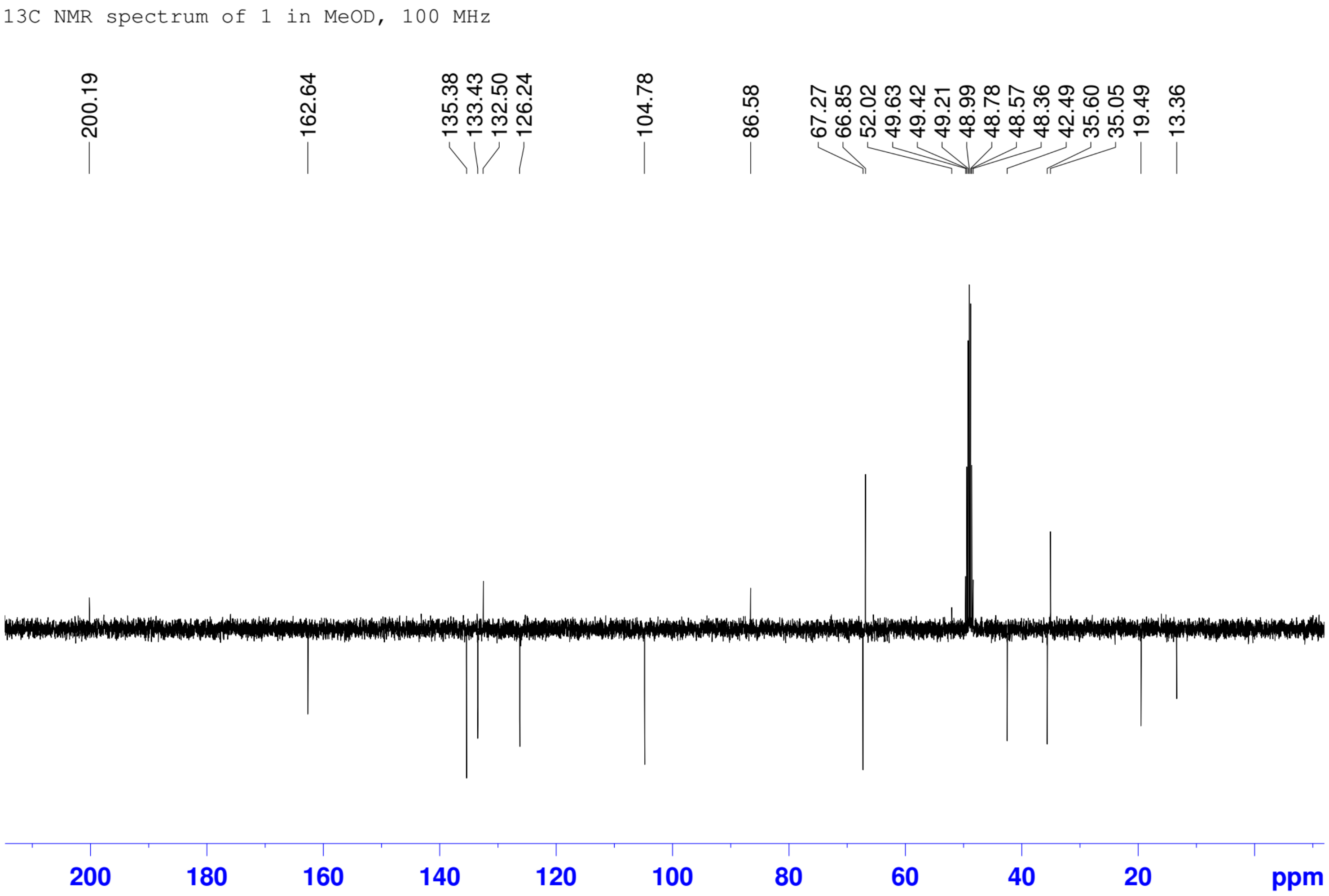

Figure S1-3. ^{13}C NMR spectrum of **1** in CD_3OD (100 MHz).

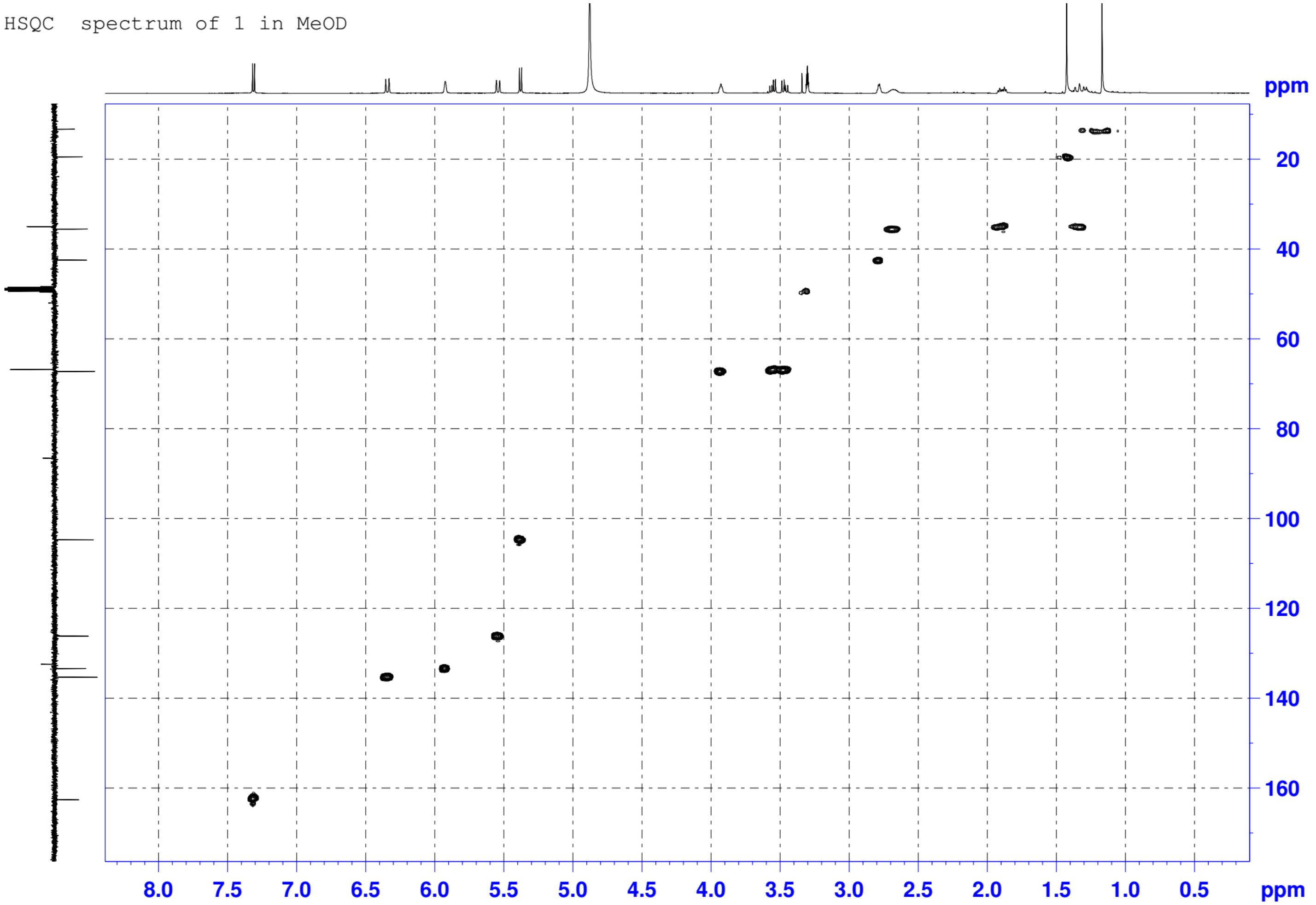

Figure S1-4. HSQC spectrum of **1** in CD_3OD.

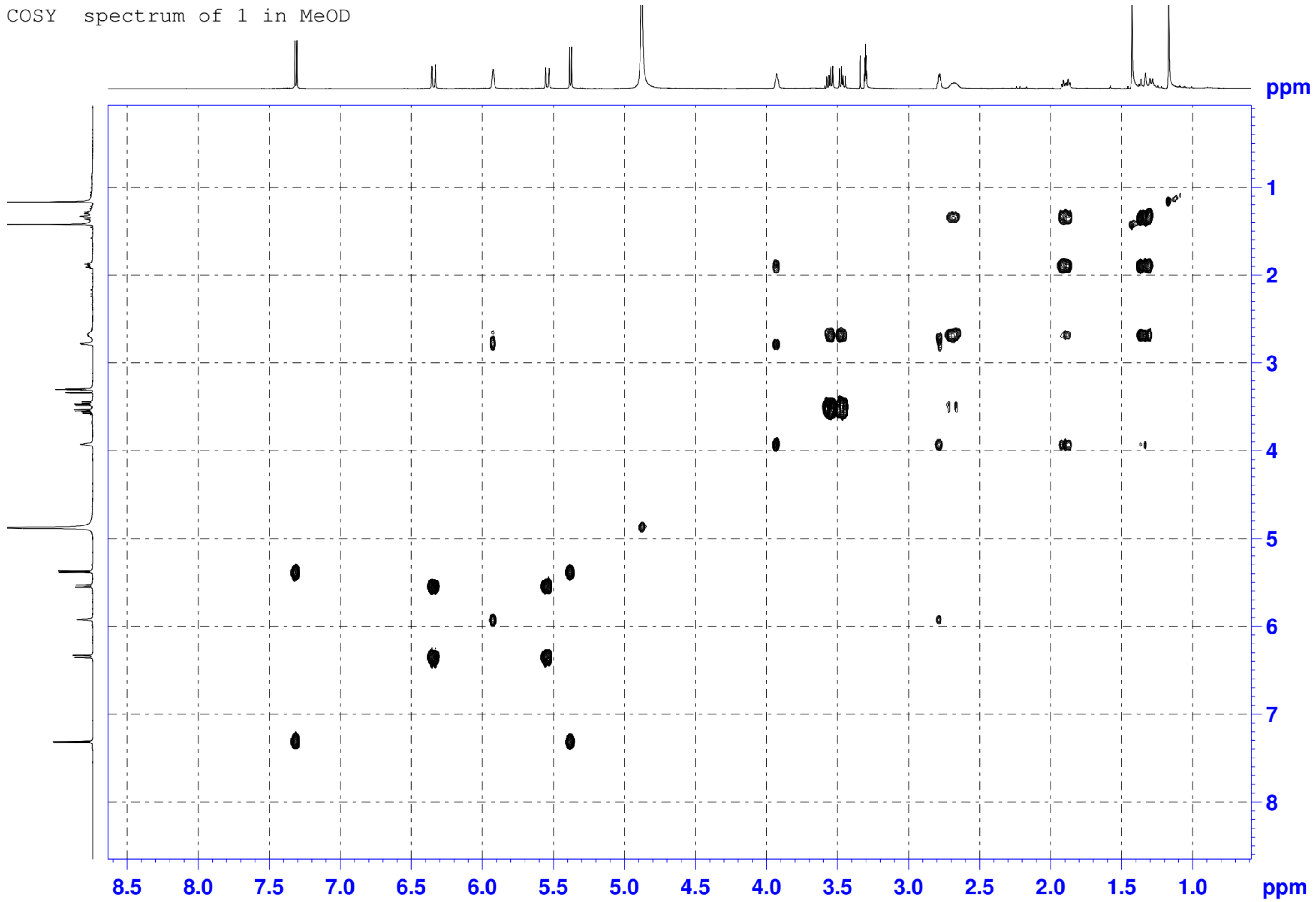

Figure S1-5. COSY spectrum of **1** in CD_3OD.

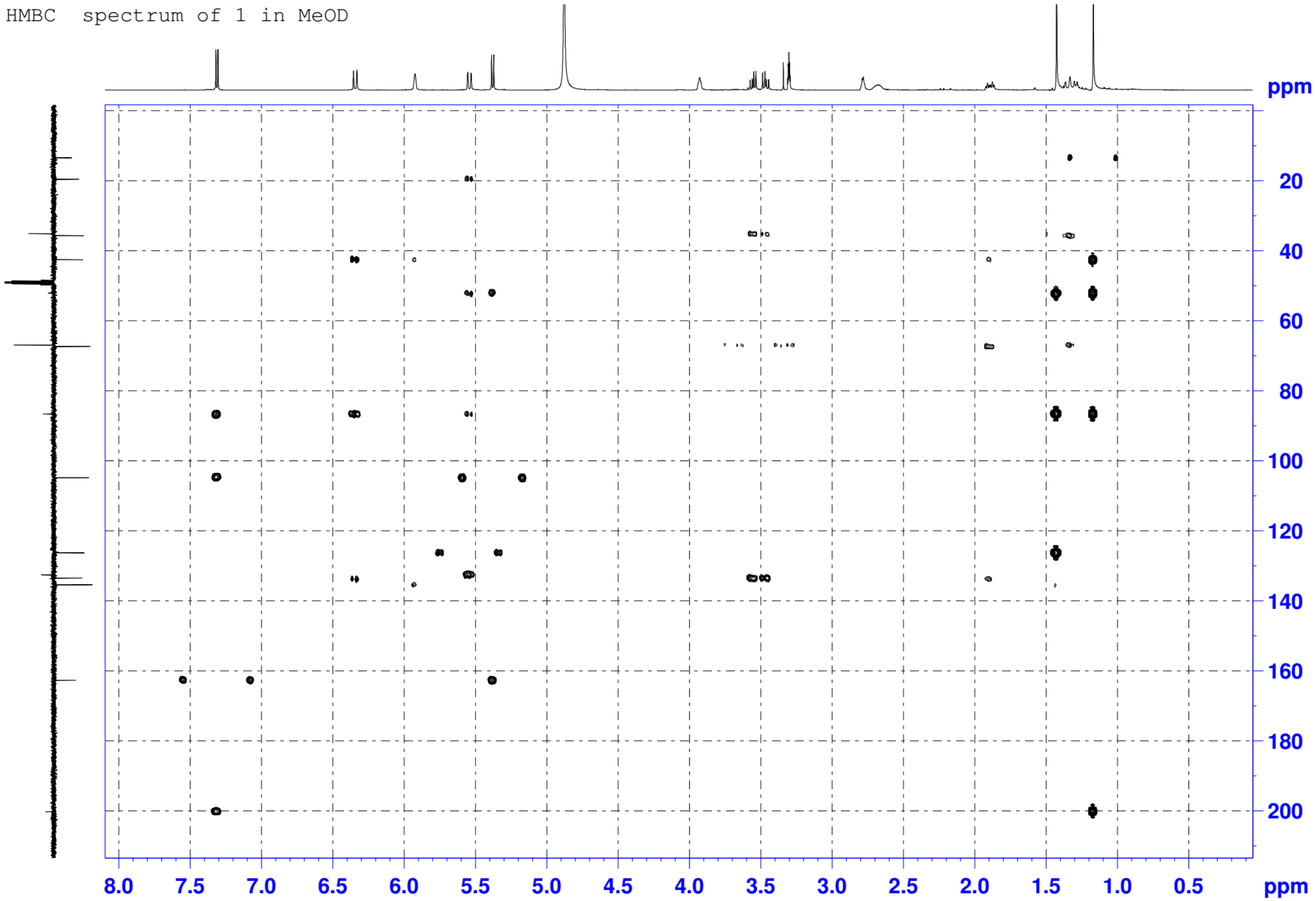

Figure S1-6. HMBC spectrum of **1** in CD_3OD.

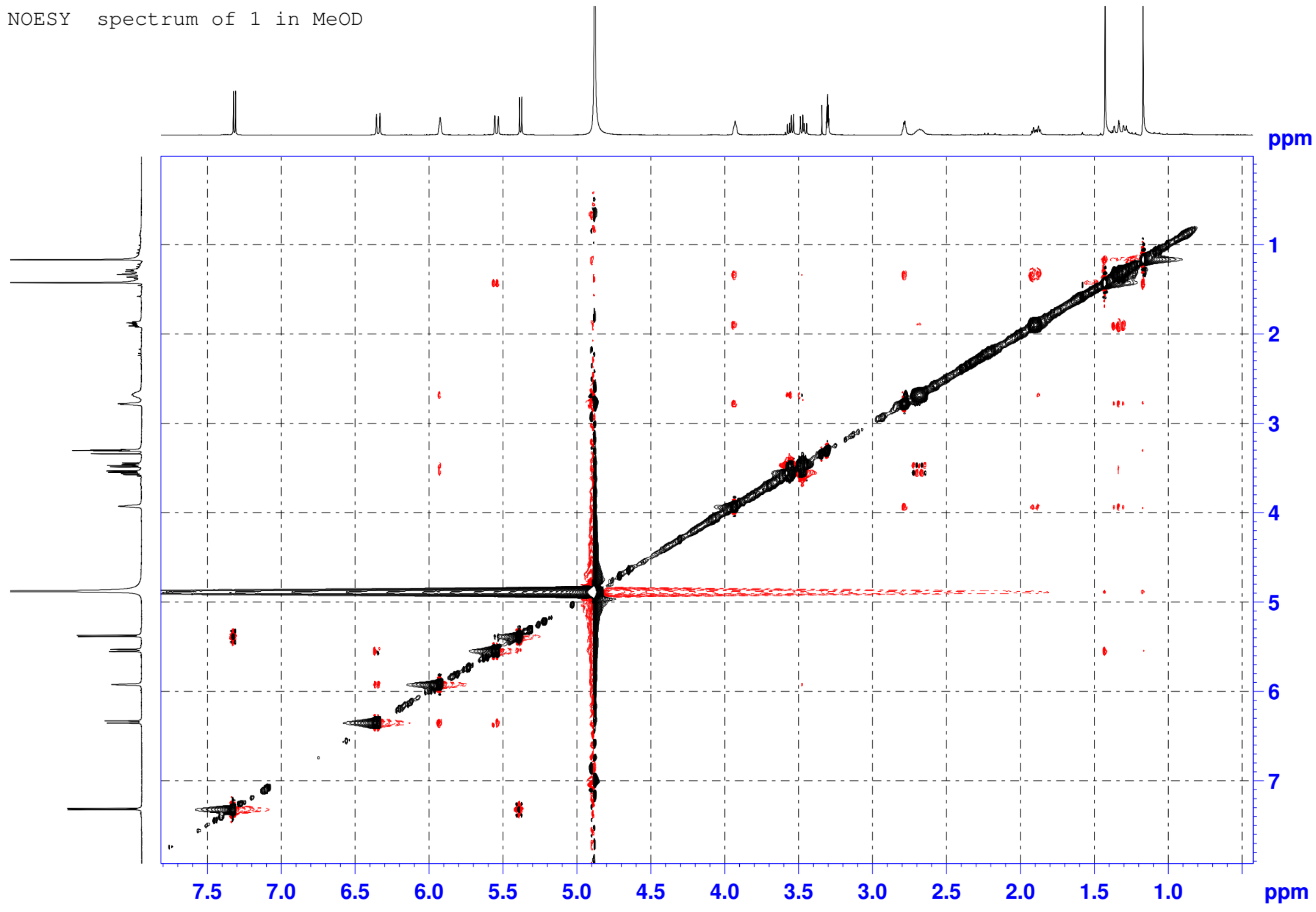

Figure S1-7. NOESY spectrum of **1** in CD_3OD.

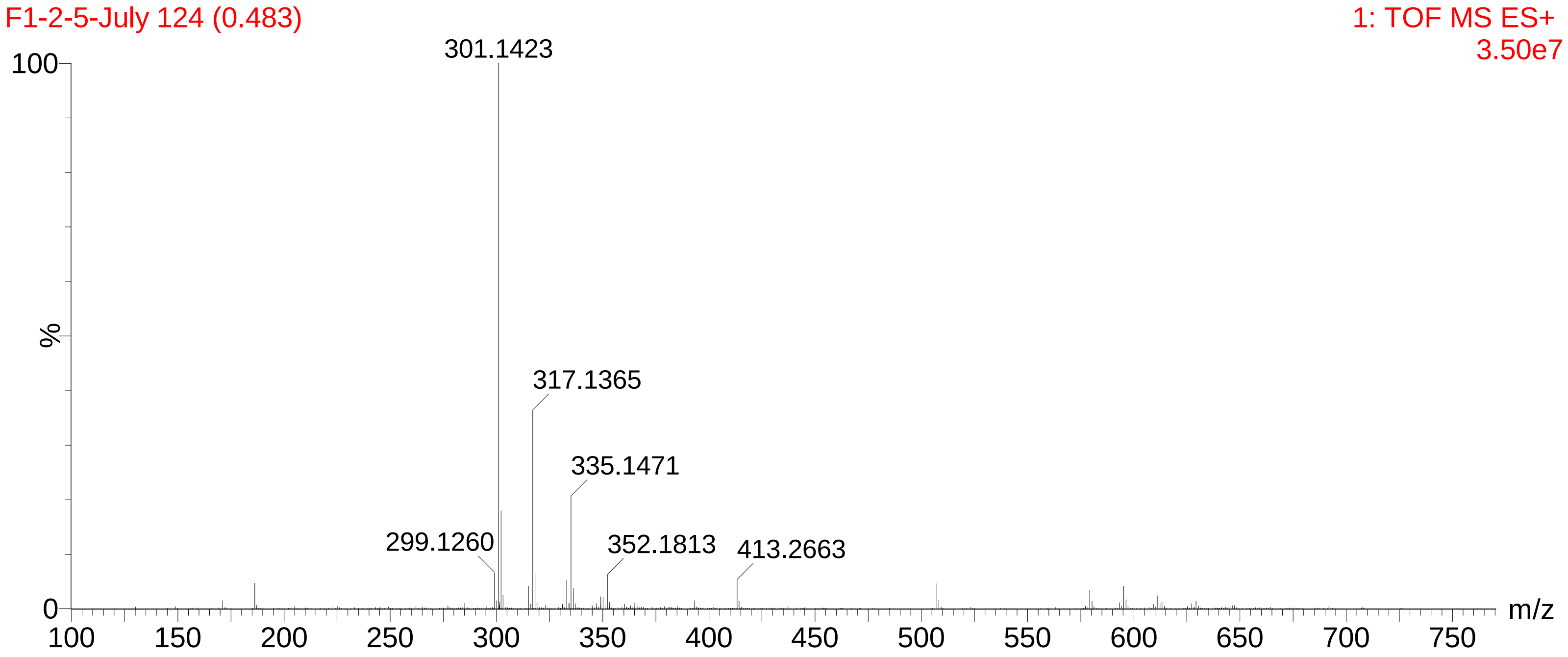

Figure S2-1. HRESIMS spectrum of **2**.

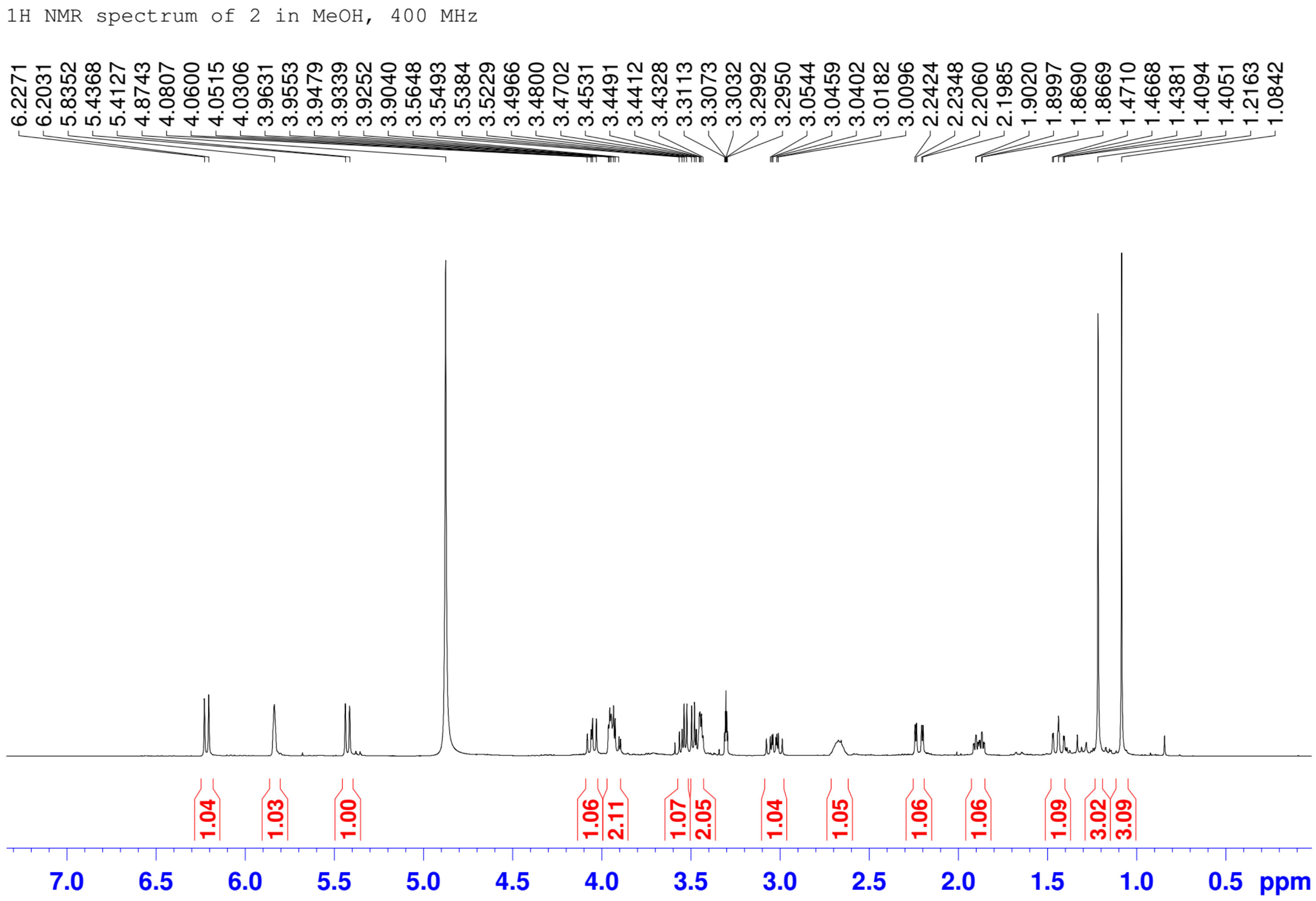

Figure S2-2. ^{1}H NMR spectrum of **2** in CD_3OD (400 MHz).

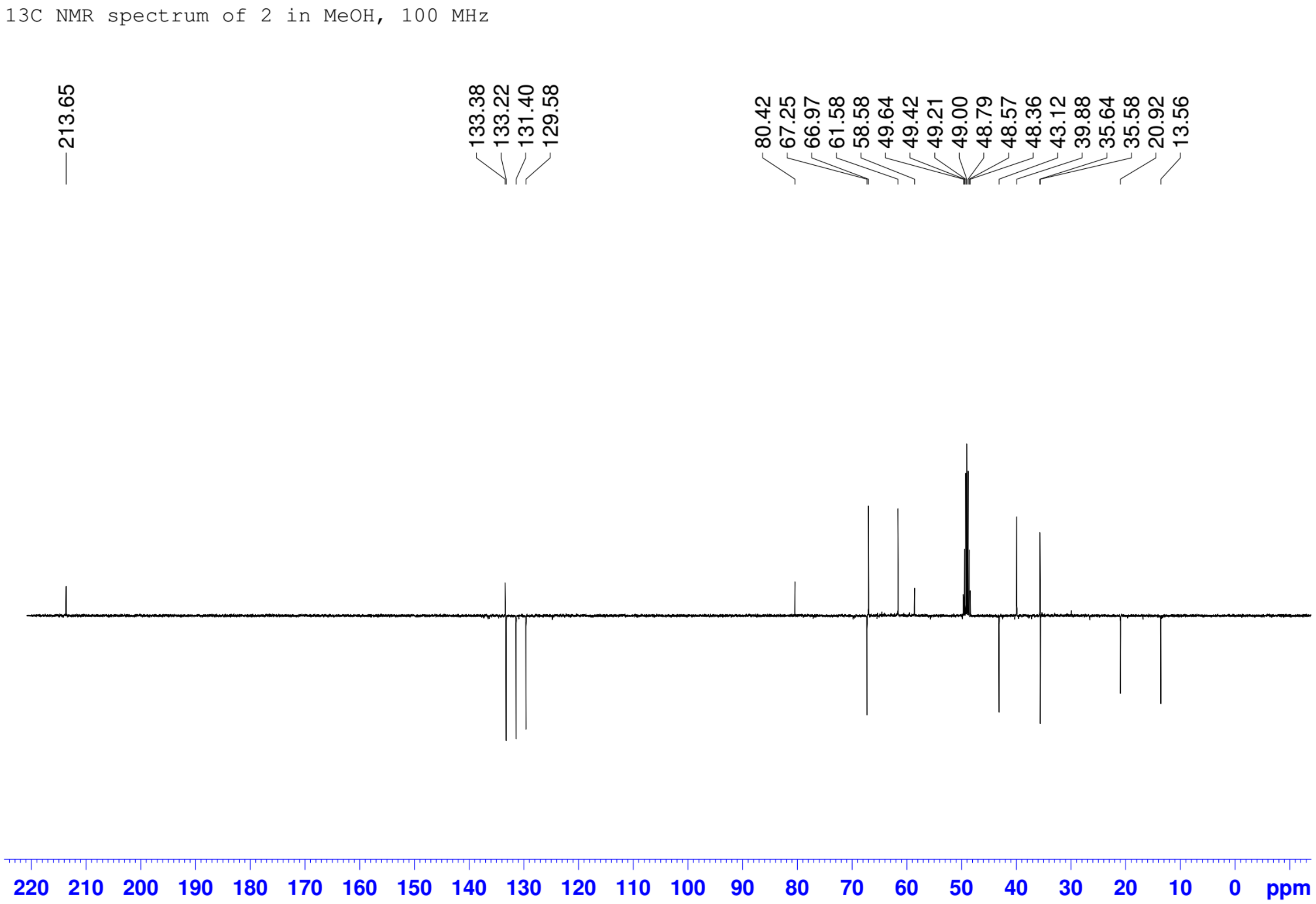

Figure S2-3. ^{13}C NMR spectrum of **3** in CD_3OD (100 MHz).

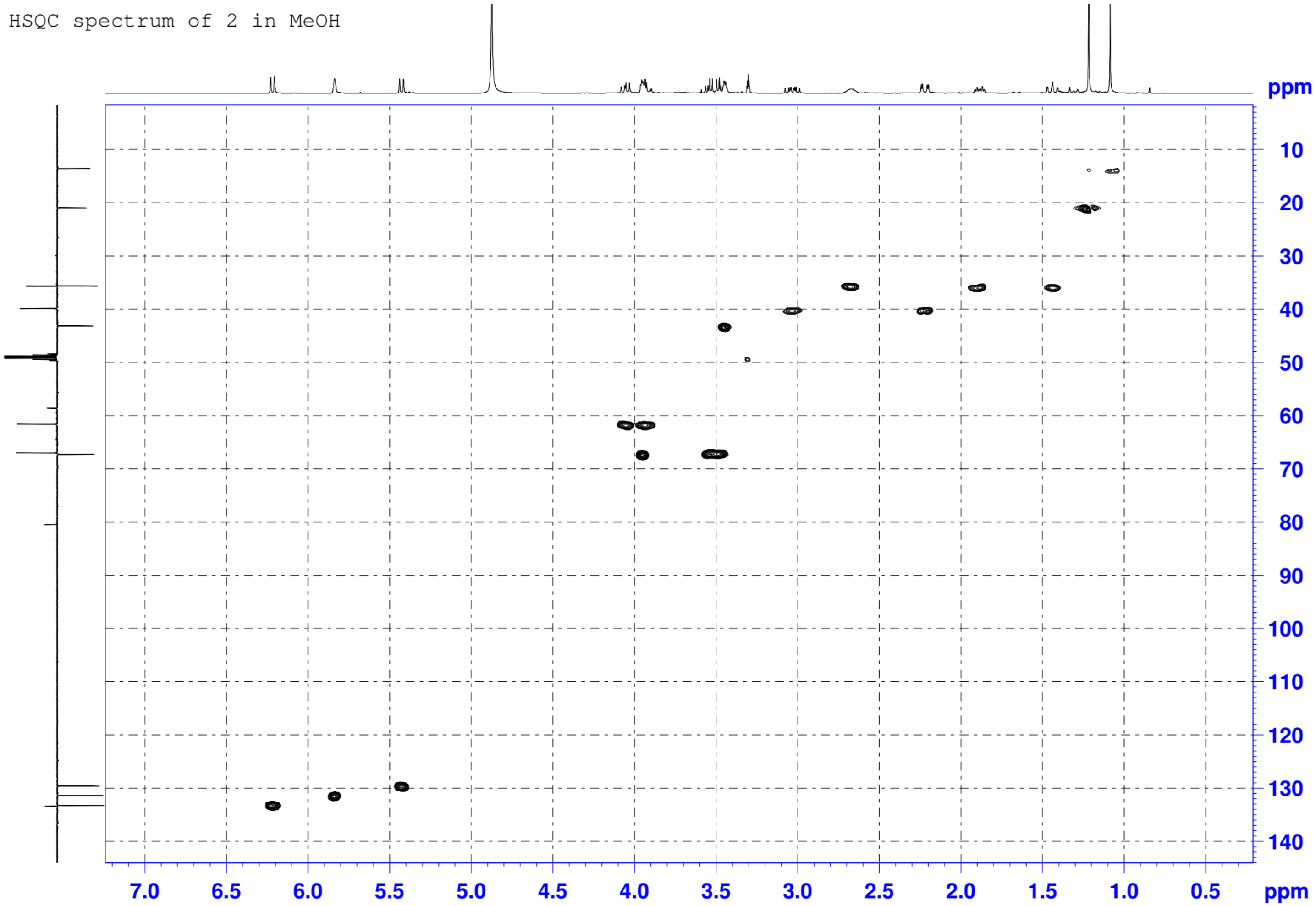

Figure S2-4. HSQC spectrum of **2** in CD_3OD.

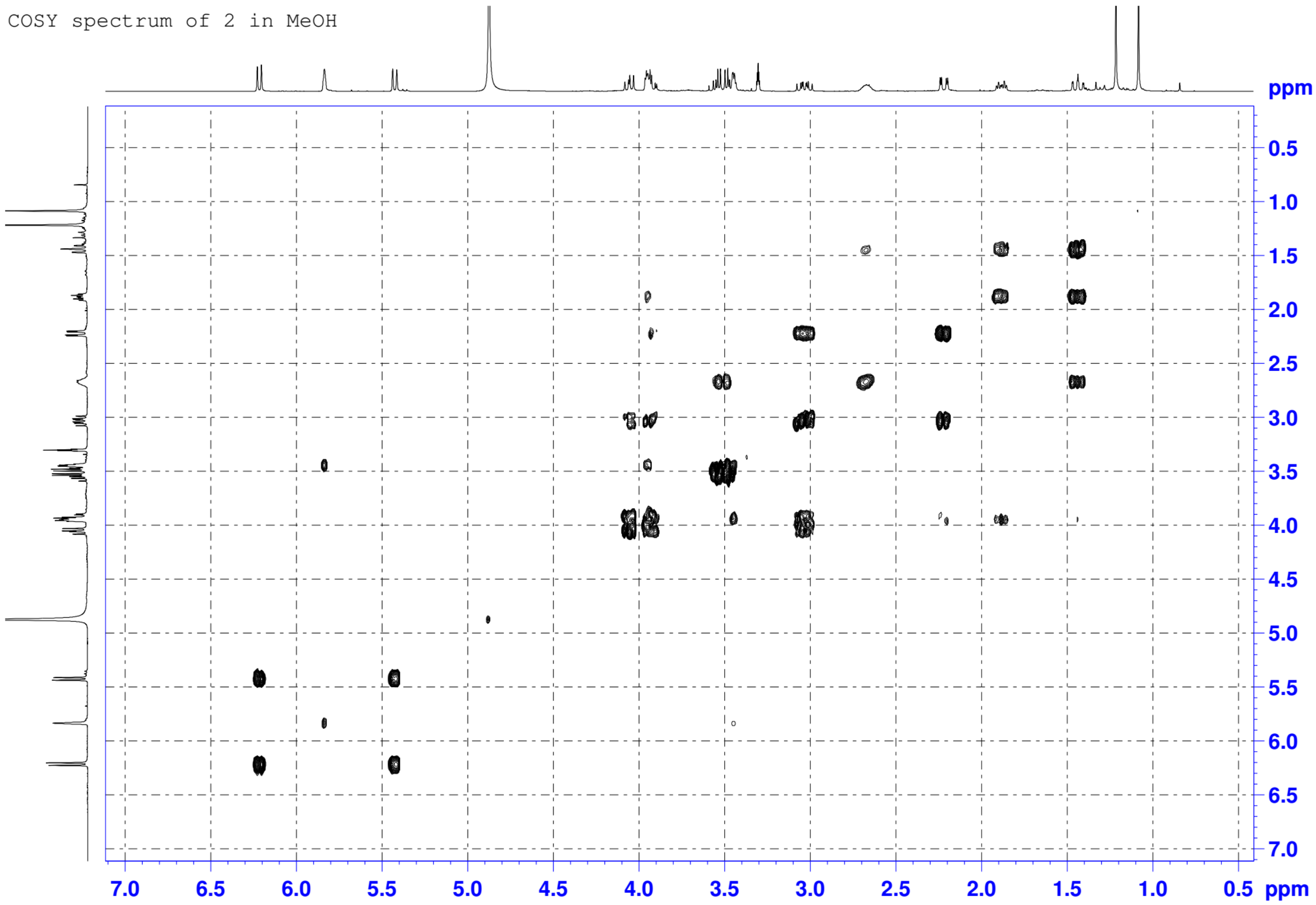

Figure S2-5. COSY spectrum of **2** in CD_3OD.

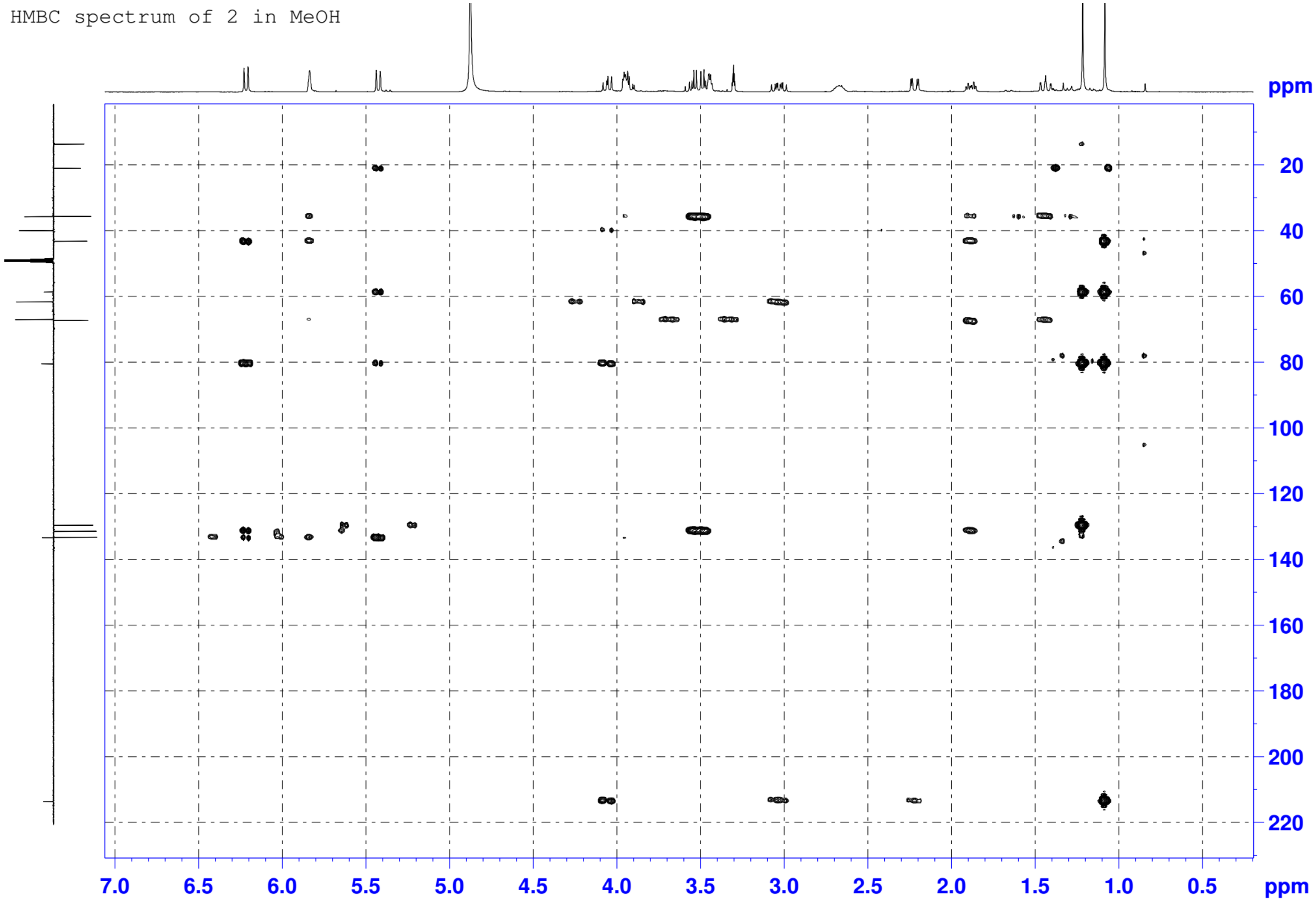

Figure S2-6. HMBC spectrum of **2** in CD_3OD.

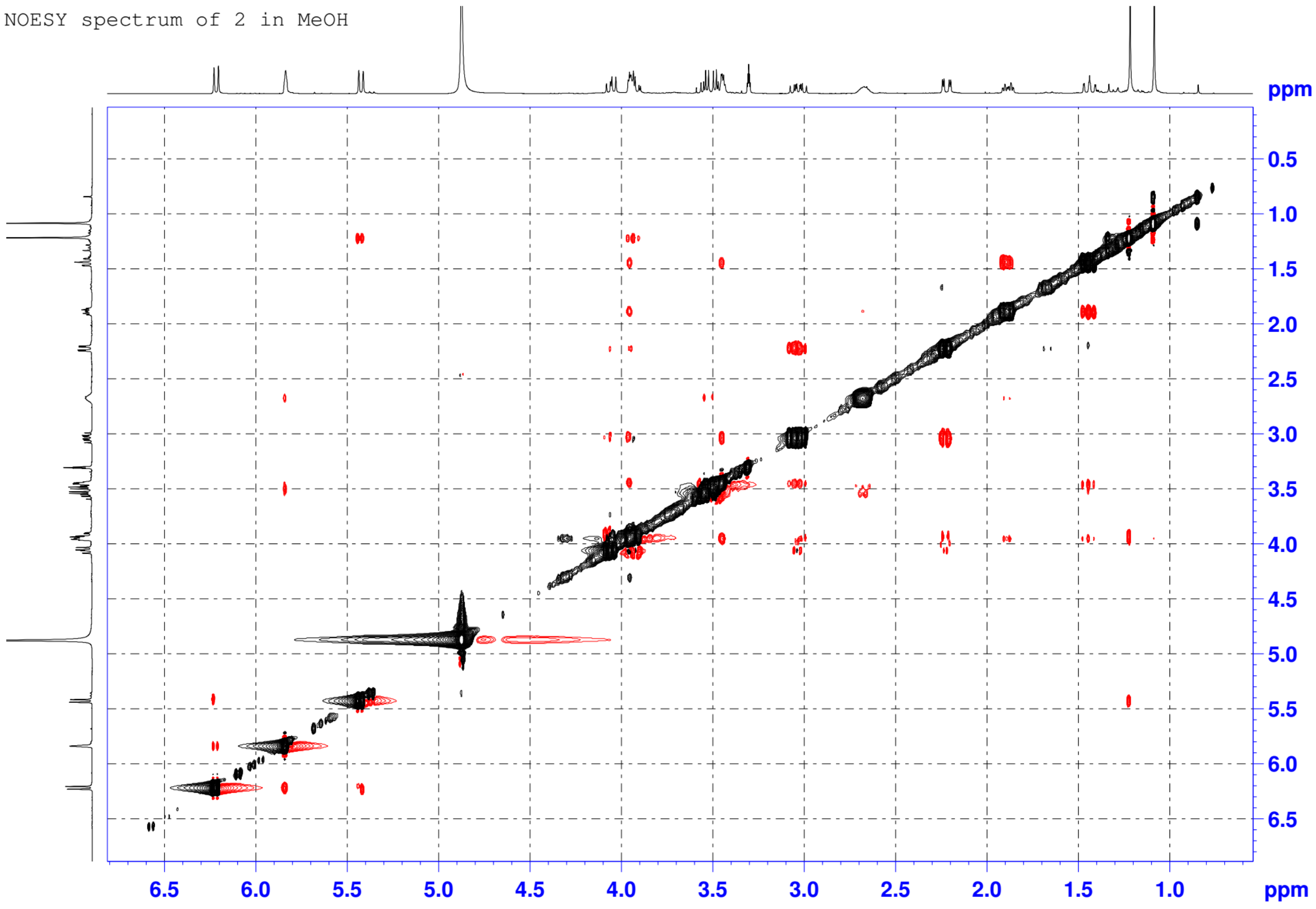

Figure S2-7. NOESY spectrum of **2** in CD_3OD.

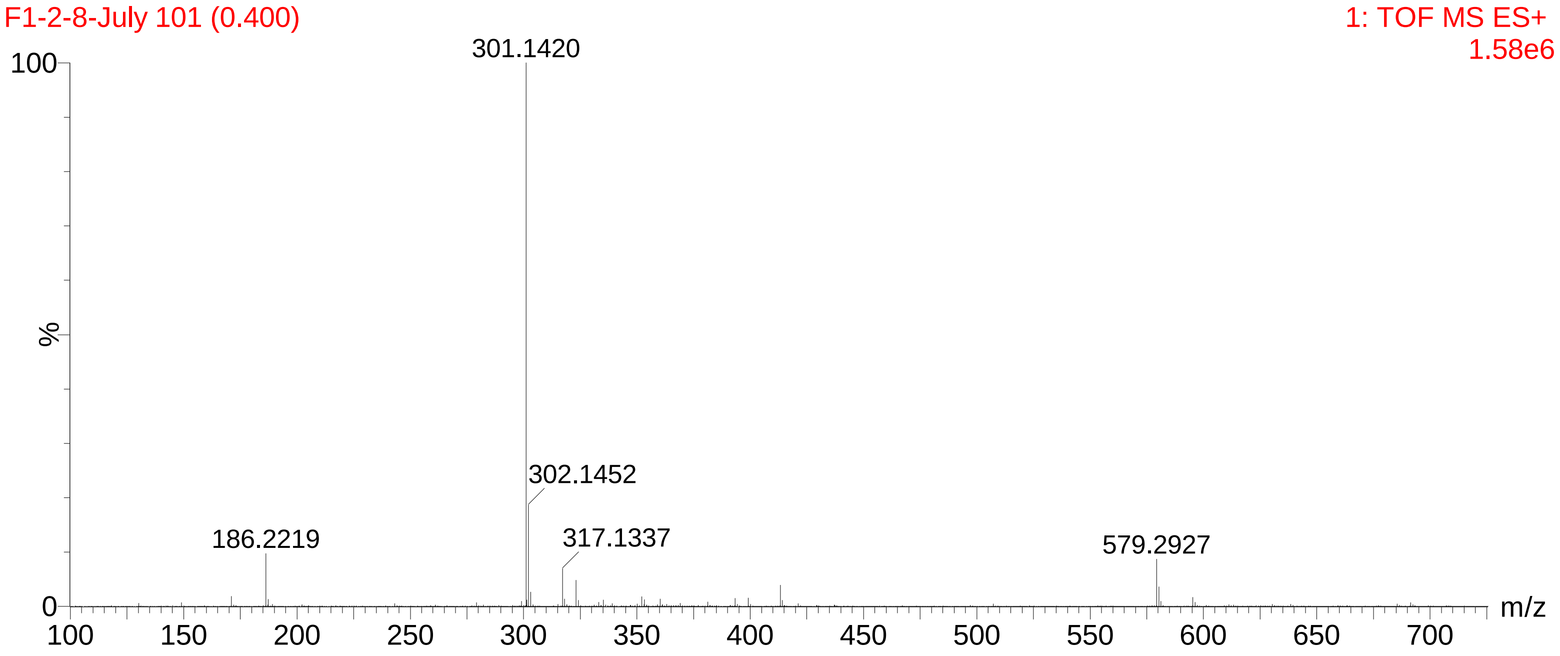

Figure S3-1. HRESIMS spectrum of **3**.

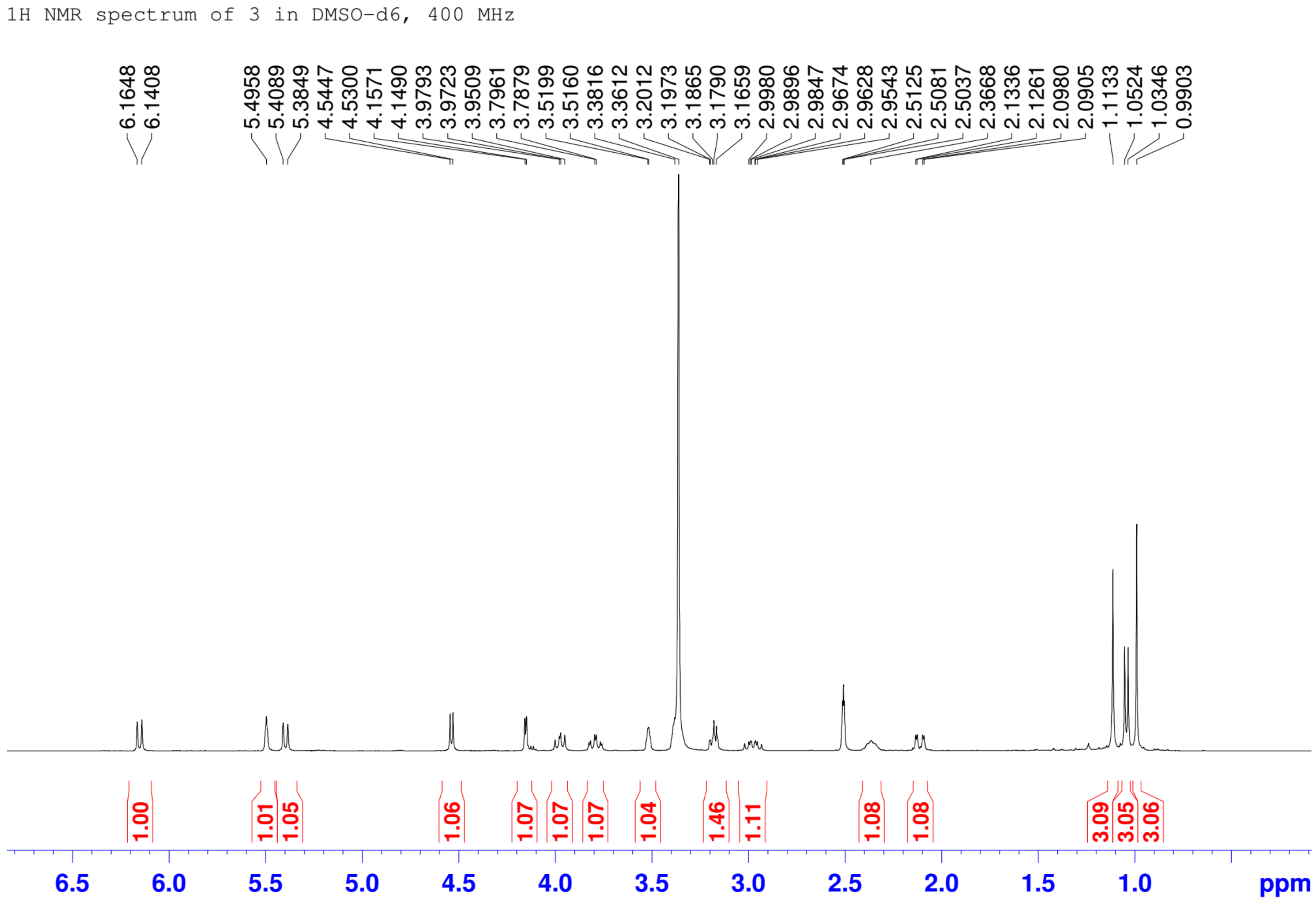

Figure S3-2. ^{1}H NMR spectrum of **3** in DMSO-d_6 (400 MHz).

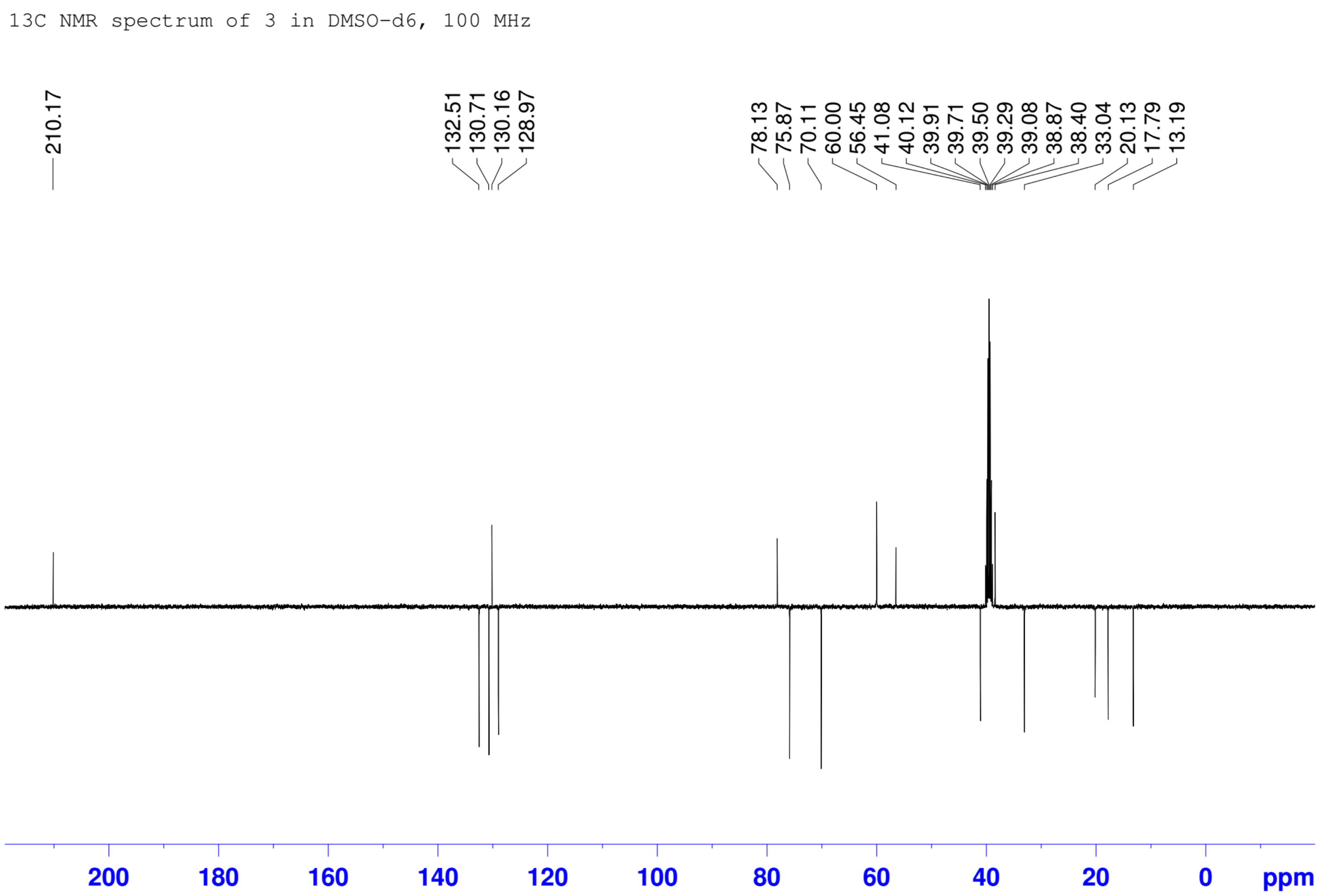

Figure S3-3. ^{13}C NMR spectrum of **3** in DMSO-d_6 (100 MHz).

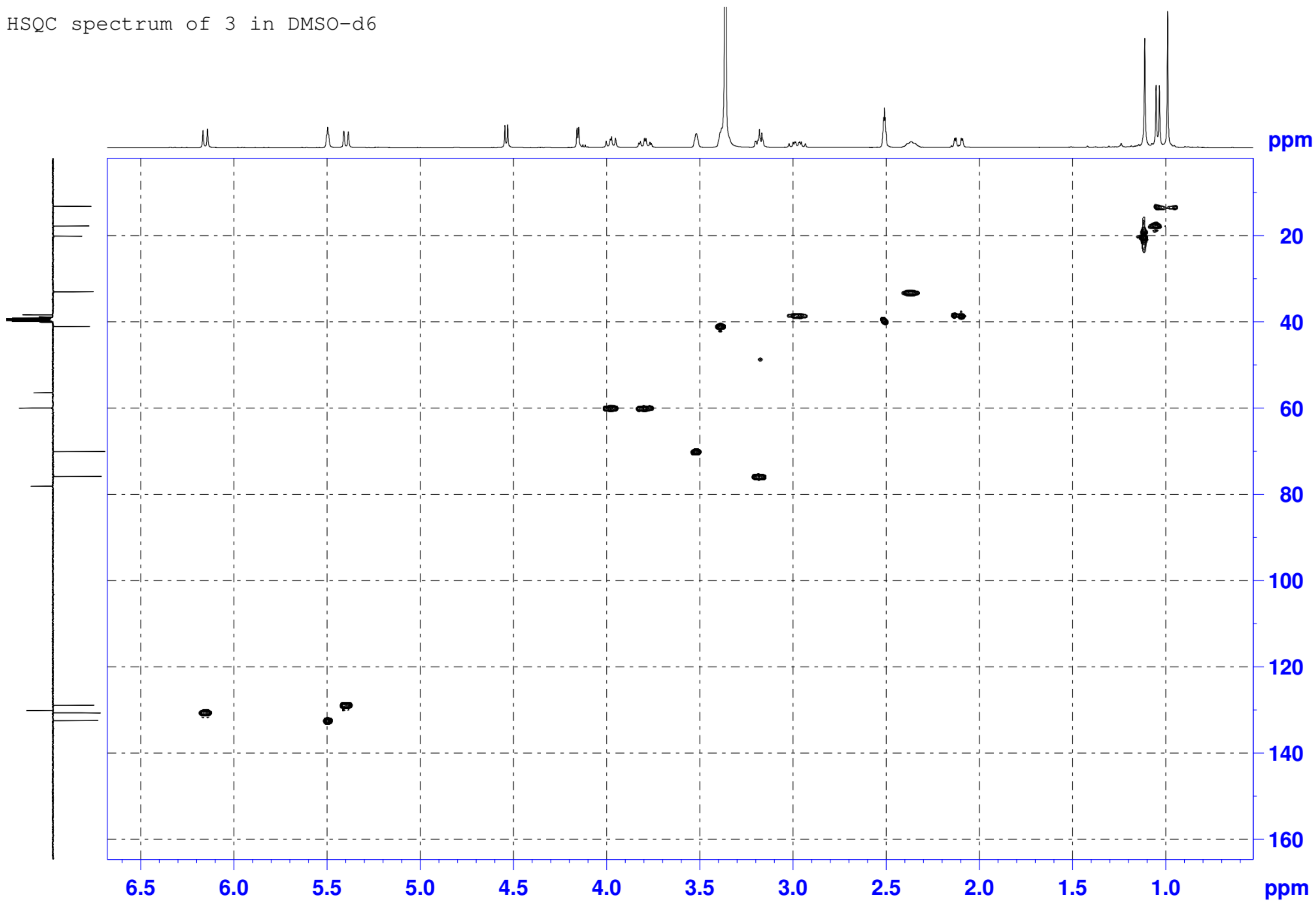

Figure S3-4. HSQC spectrum of **3** in DMSO-d_6.

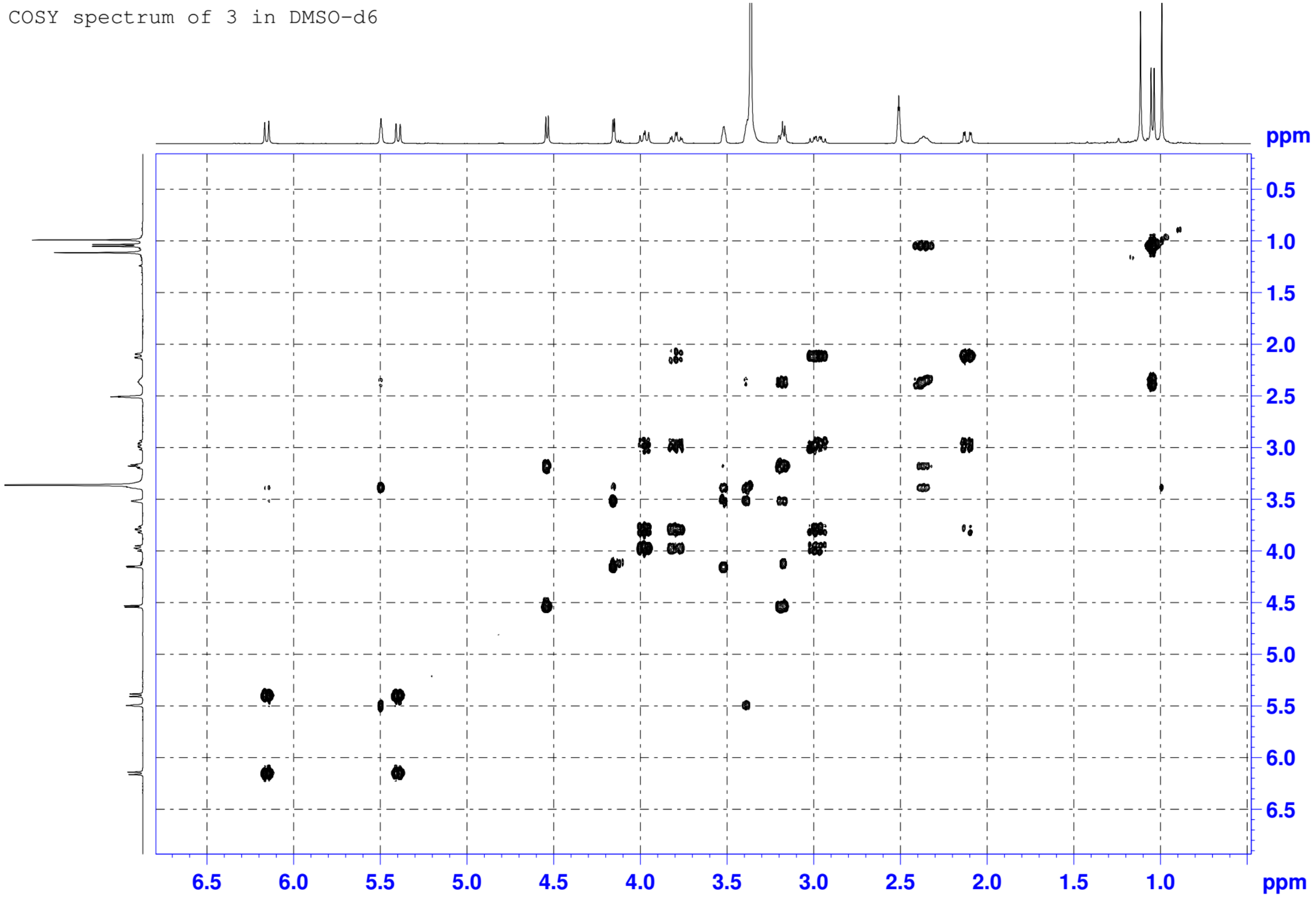

Figure S3-5. COSY spectrum of **3** in DMSO-d_6.

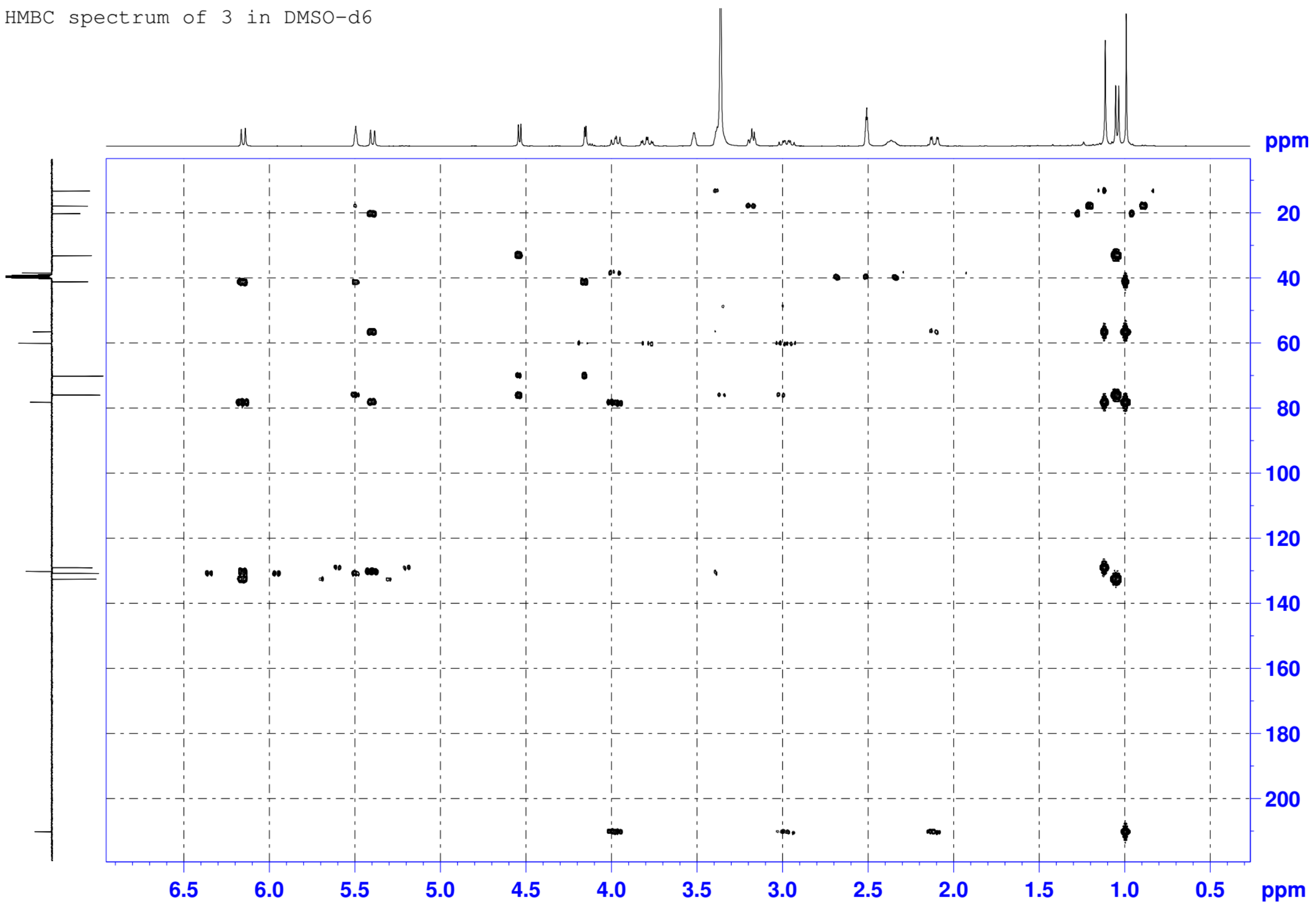

Figure S3-6. HMBC spectrum of **3** in DMSO-d_6.

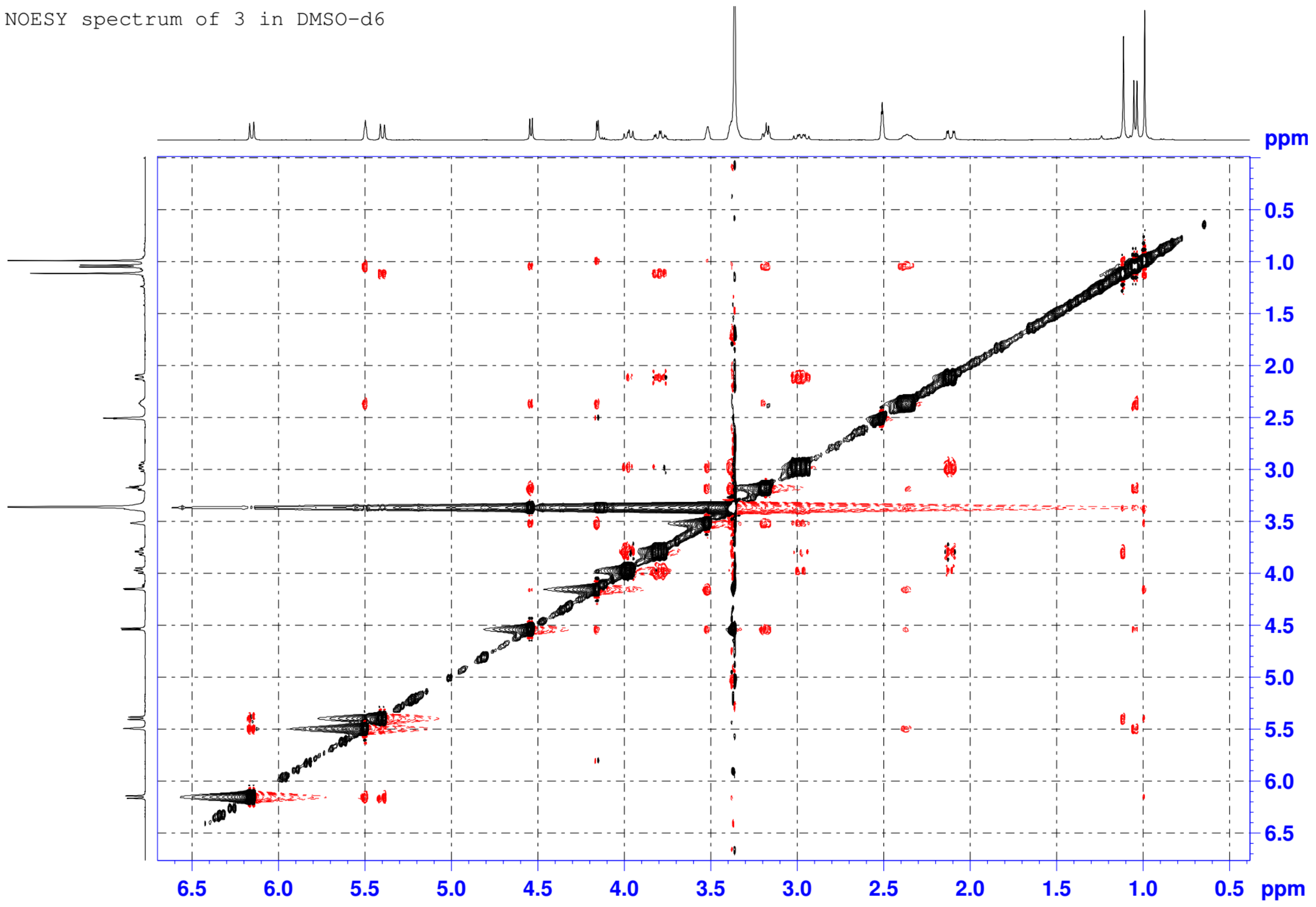

Figure S3-7. NOESY spectrum of **3** in DMSO-d_6.

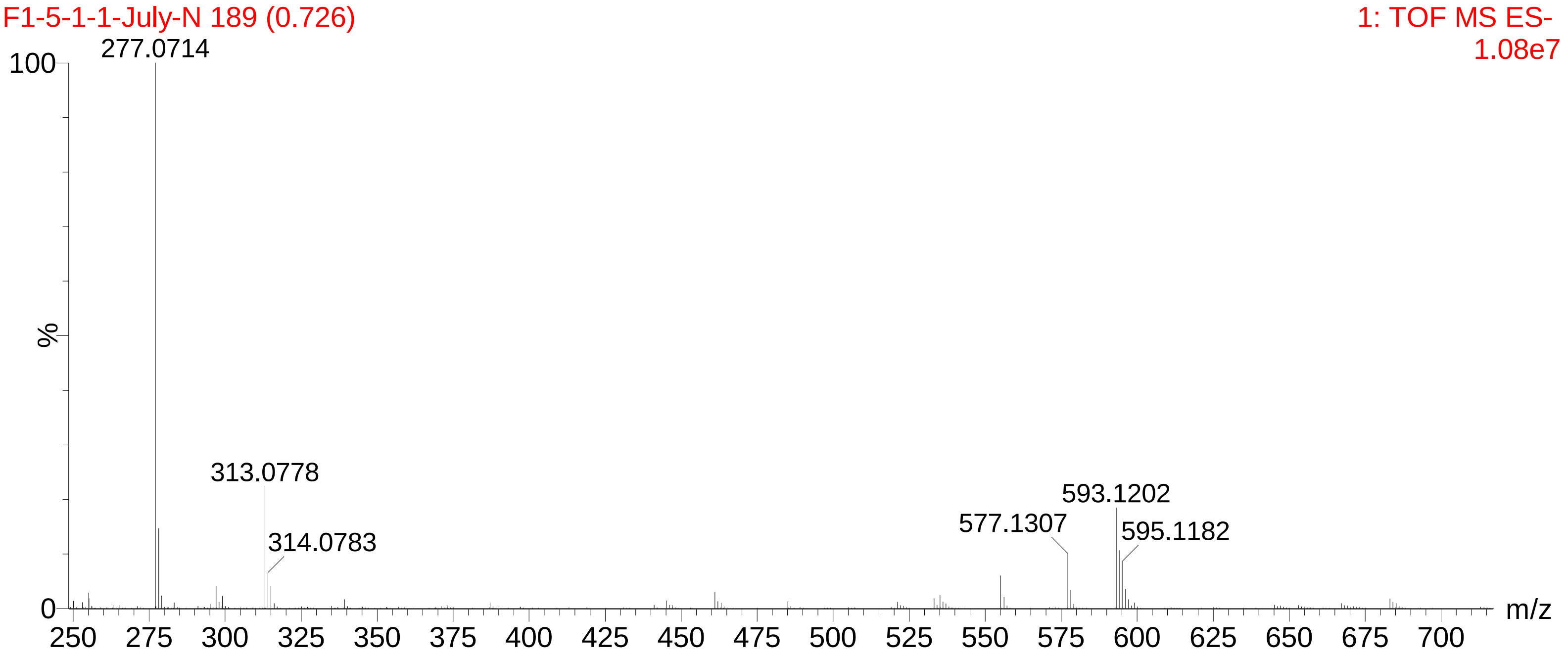

Figure S4-1. HRESIMS spectrum of **6**.

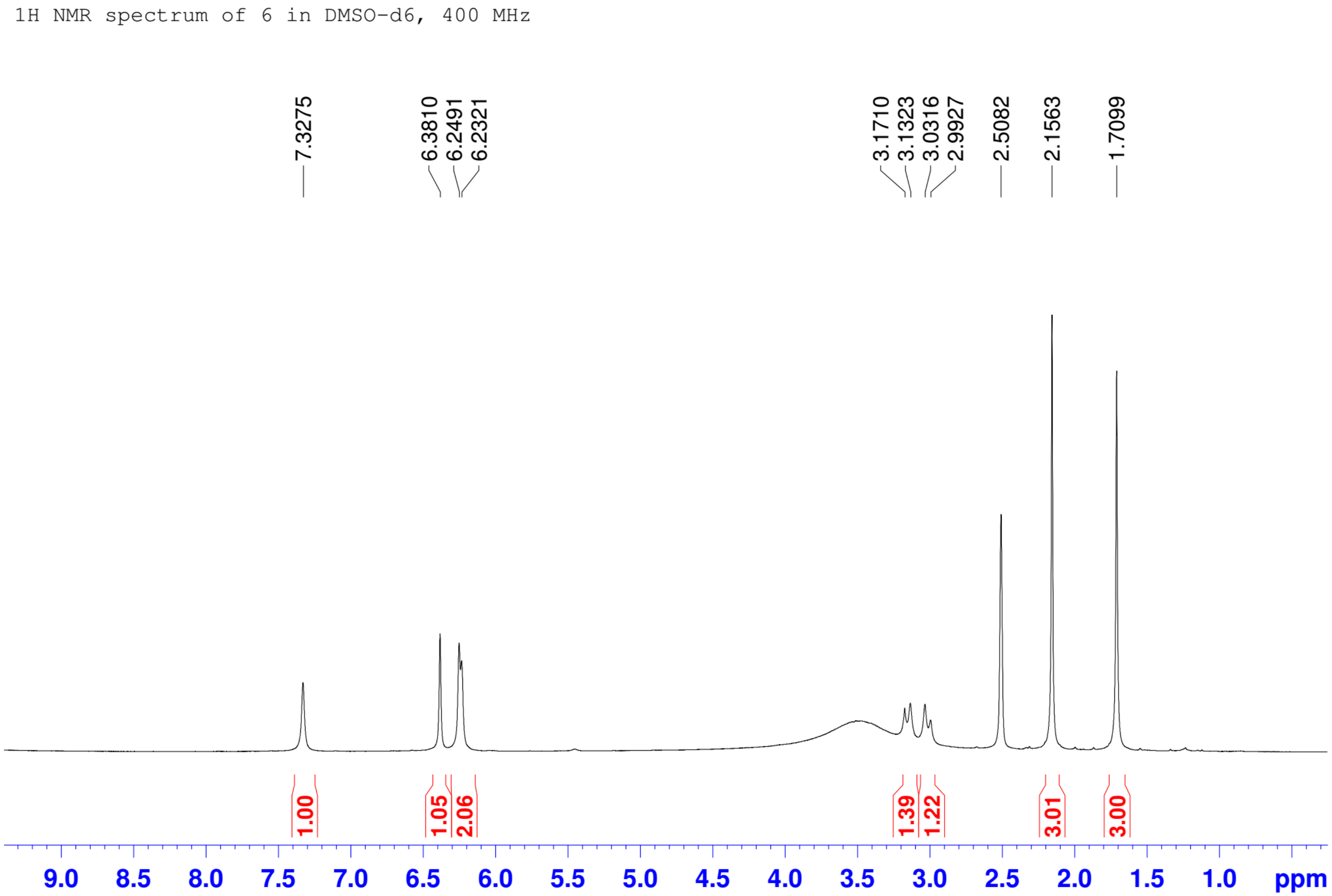

Figure S4-2. ^{1}H NMR spectrum of **6** in DMSO-d_6 (400 MHz).

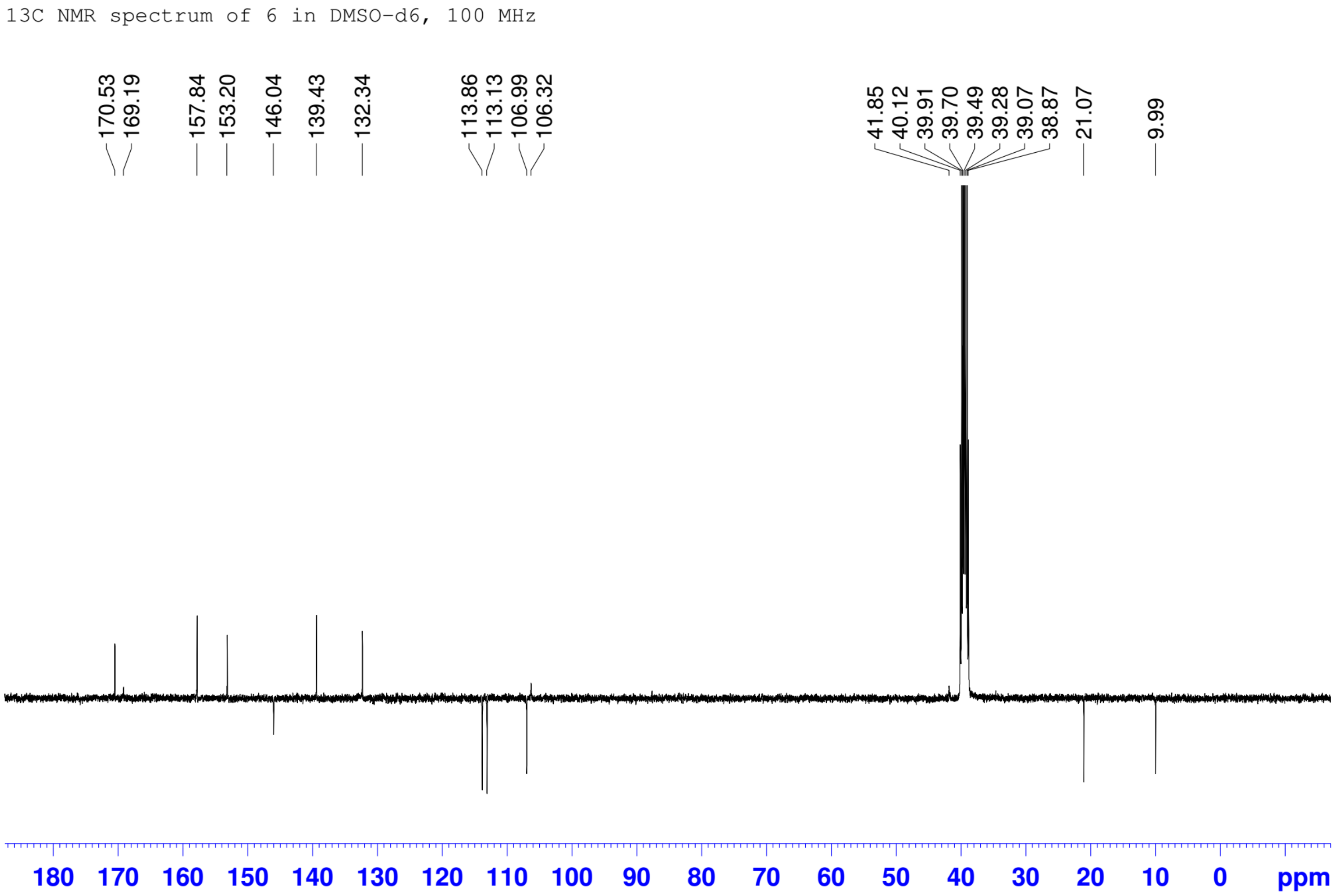

Figure S4-3. ${}^{13}C$ NMR spectrum of **6** in DMSO-d_6 (100 MHz).

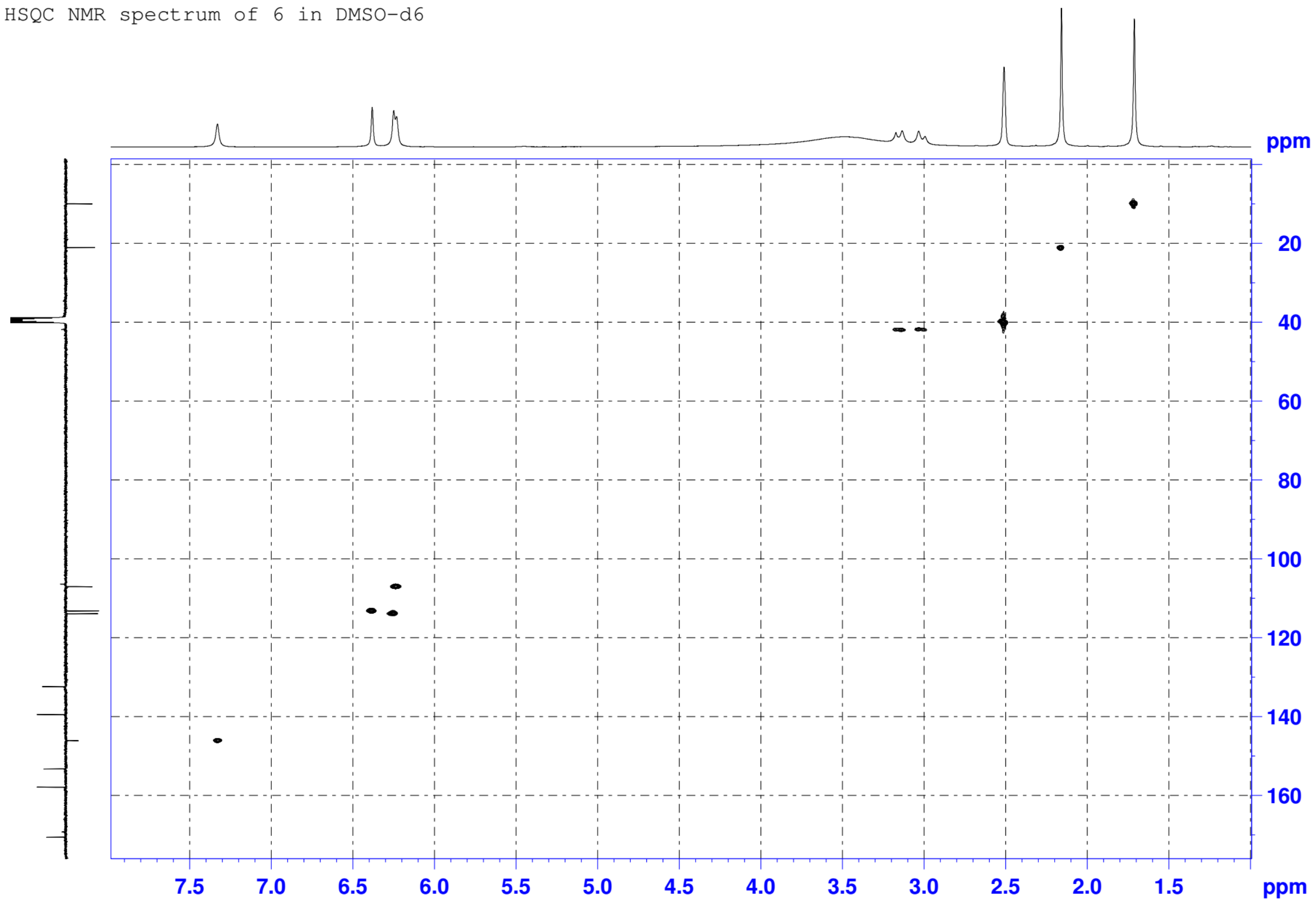

Figure S4-4. HSQC spectrum of **6** in DMSO-d_6.

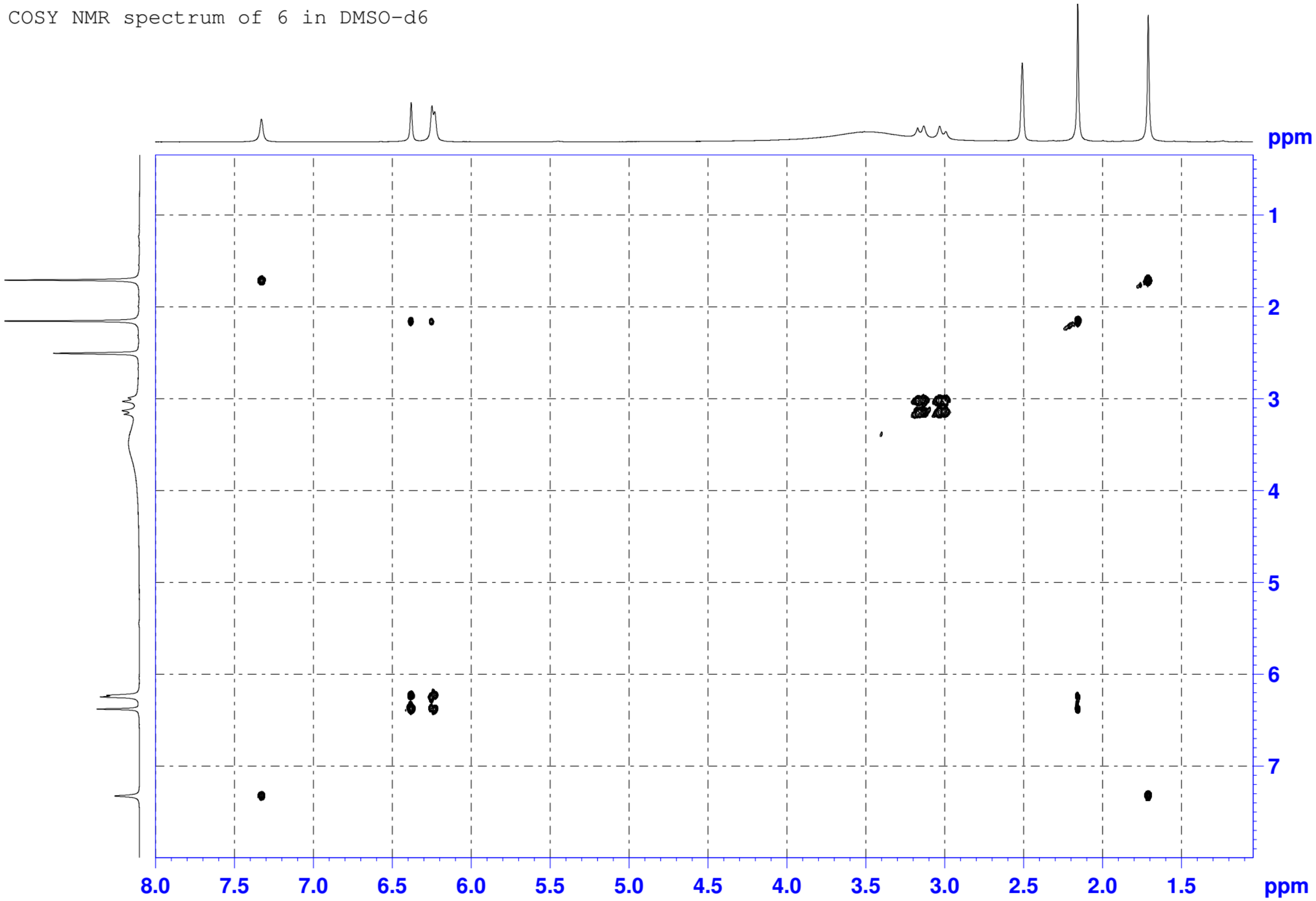

Figure S4-5. COSY spectrum of **6** in DMSO-d_6.

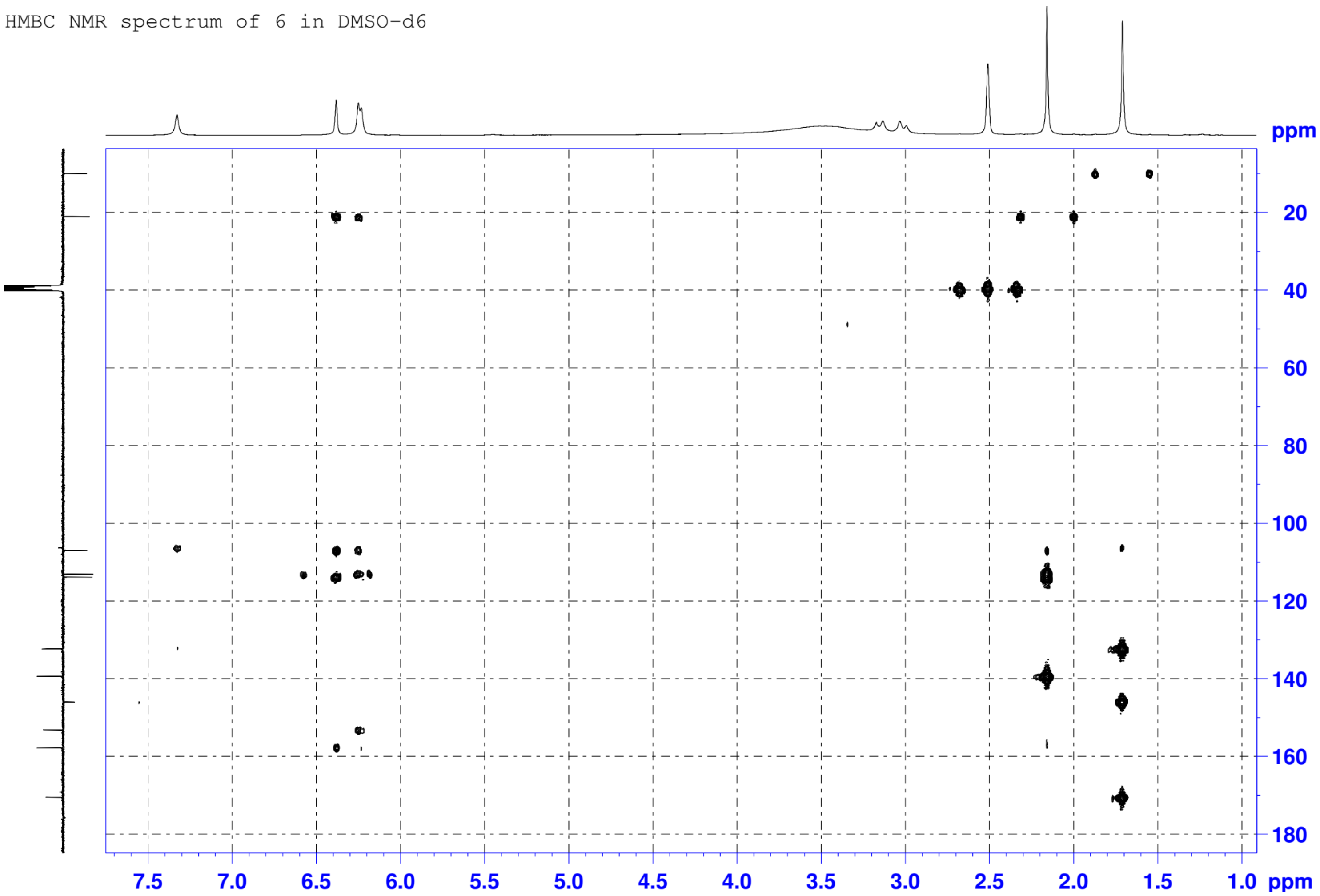

Figure S4-6. HMBC spectrum of **6** in DMSO-d_6.

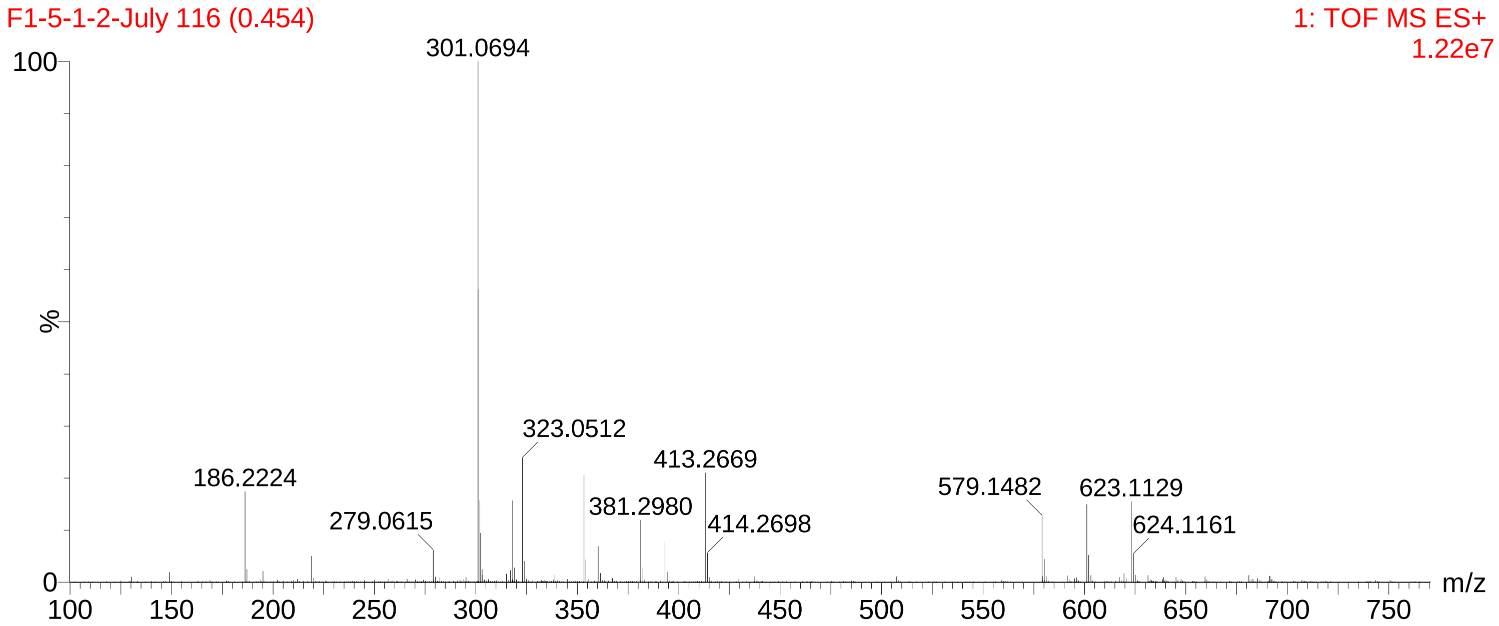

Figure S5-1. HRESIMS spectrum of **7**.

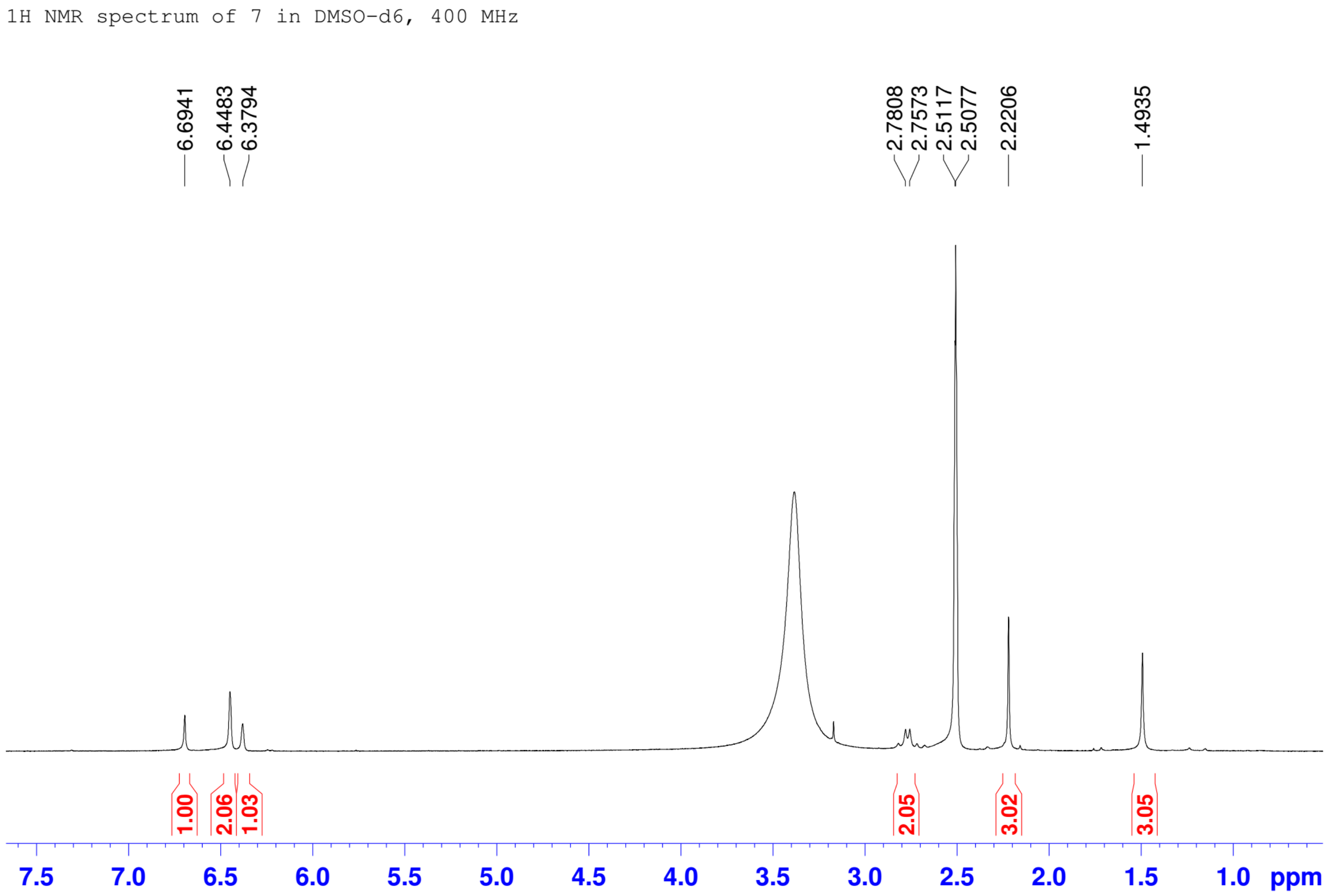

Figure S5-2. ^{1}H NMR spectrum of **7** in DMSO-d_6 (400 MHz).

13C NMR spectrum of 7 in DMSO-d6, 100 MHz

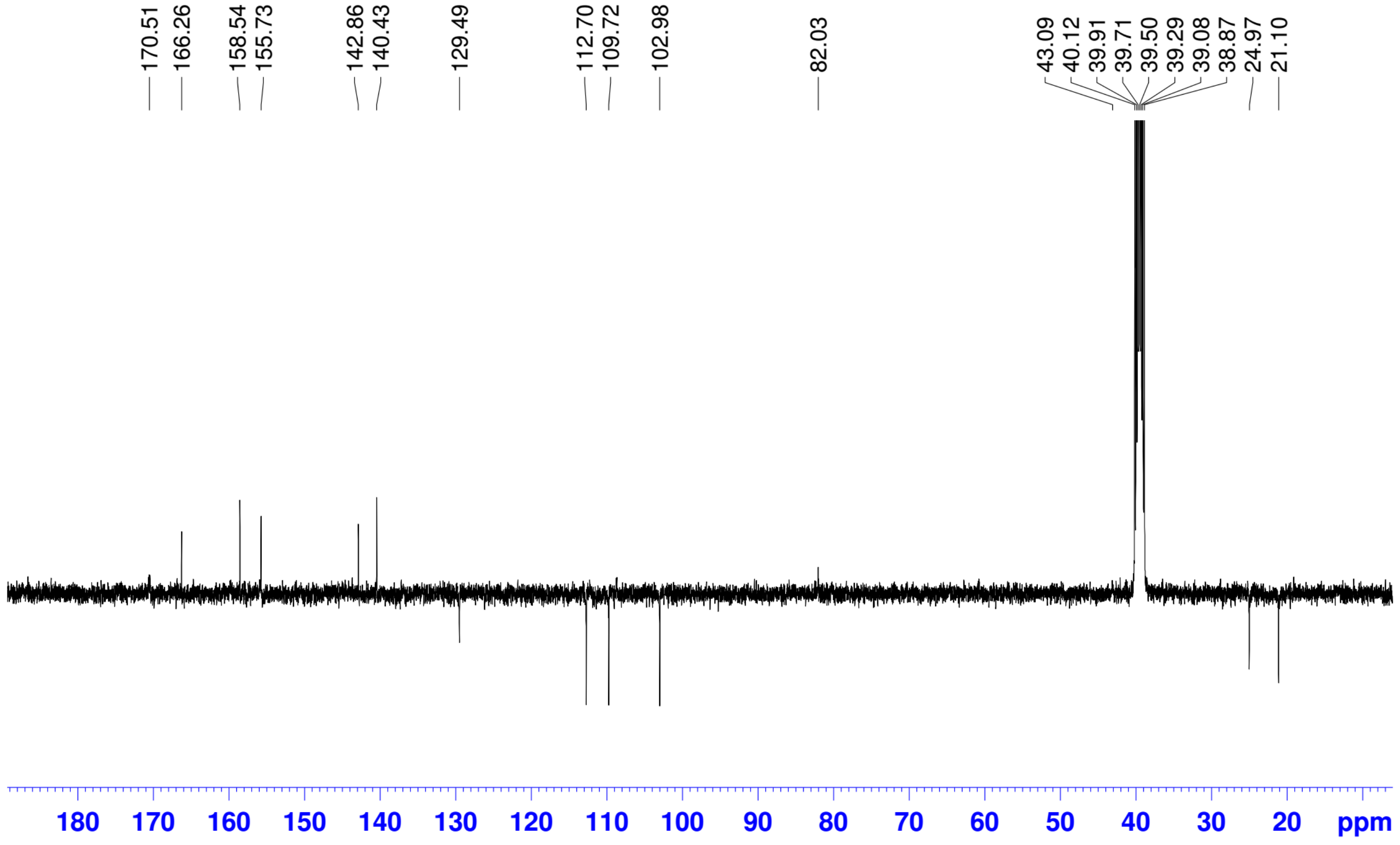

Figure S5-3. ^{13}C NMR spectrum of **7** in DMSO-d_6 (100 MHz).

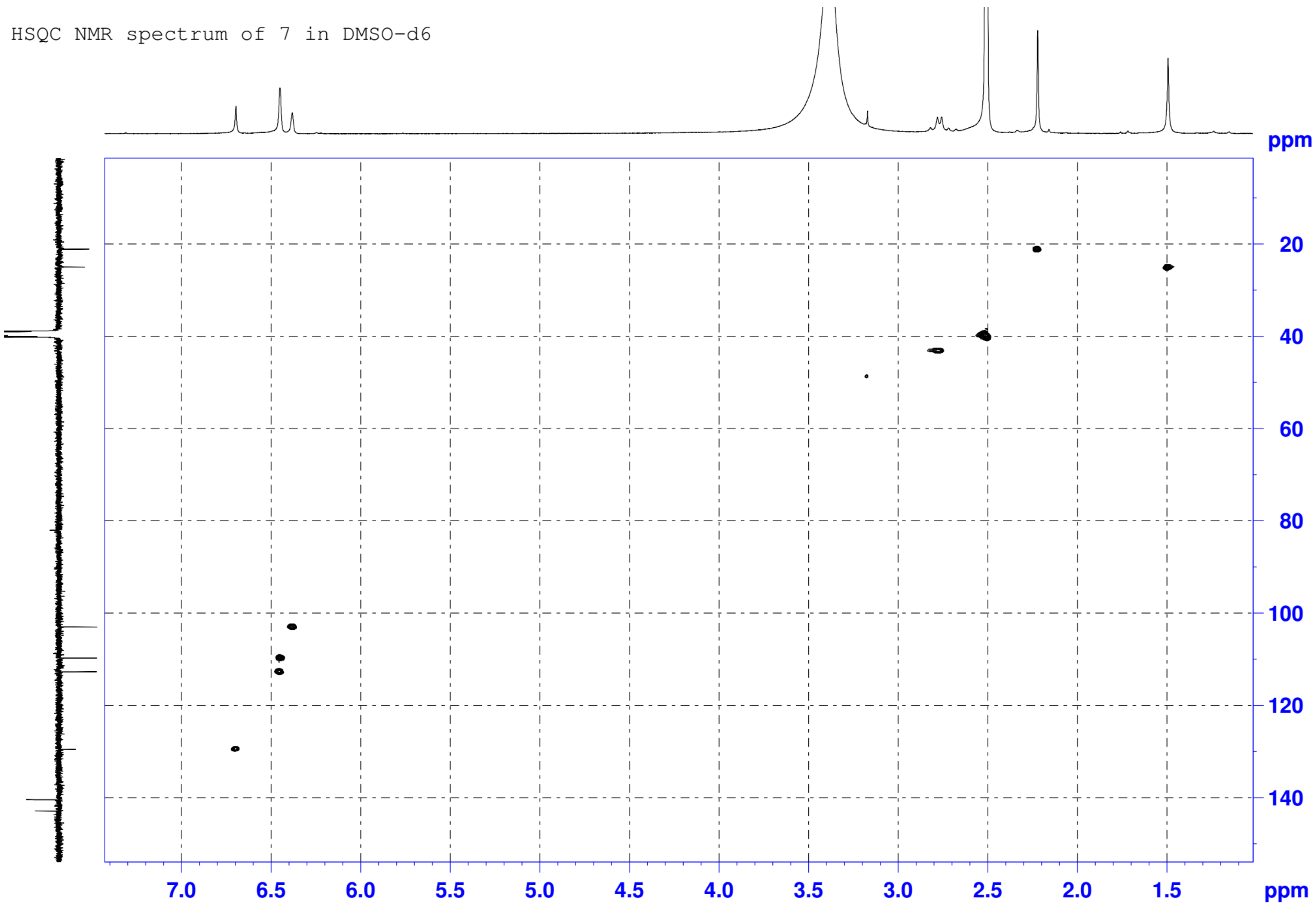

Figure S5-4. HSQC spectrum of **7** in DMSO-d_6.

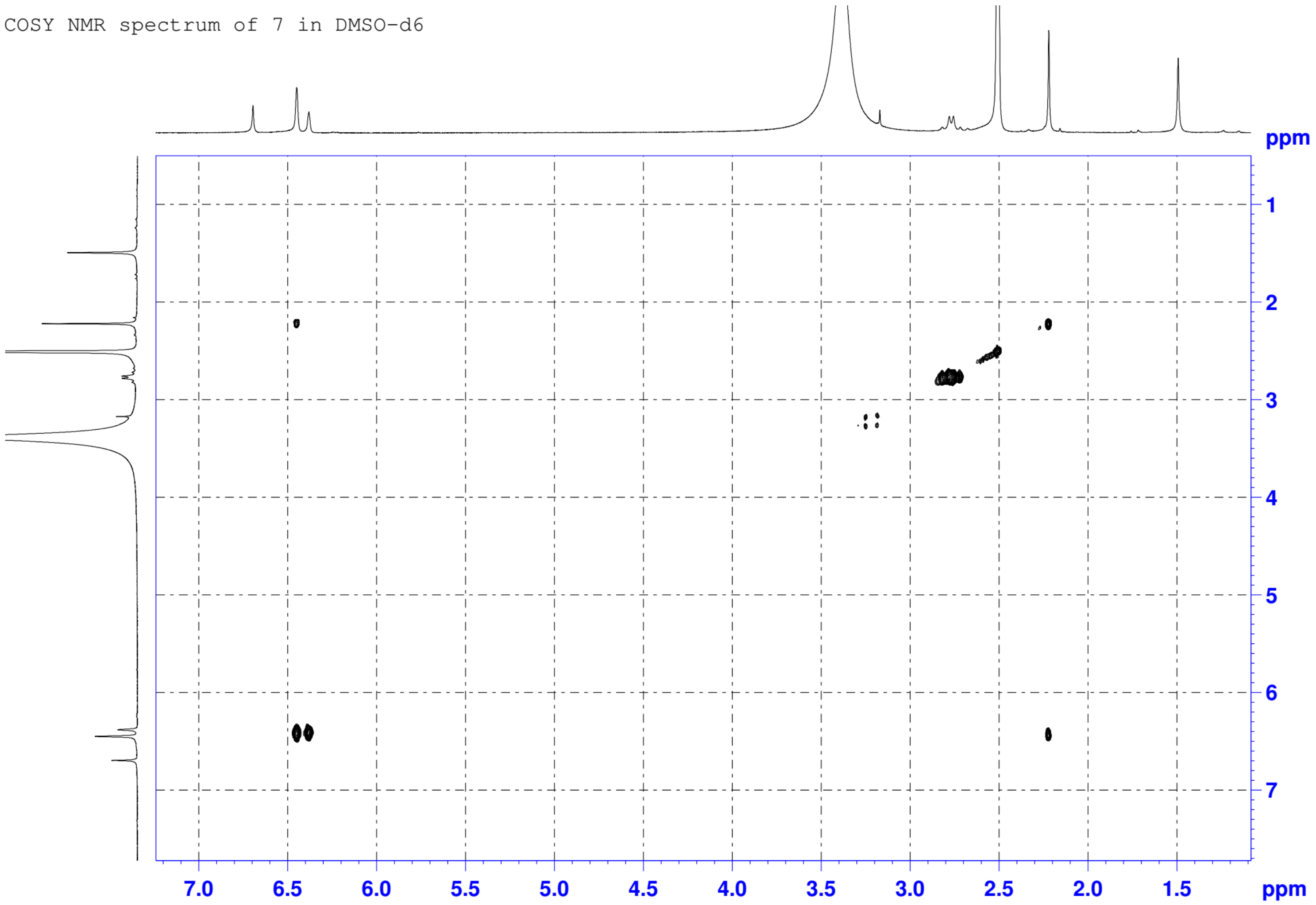

Figure S5-5. COSY spectrum of **7** in DMSO-d_6.

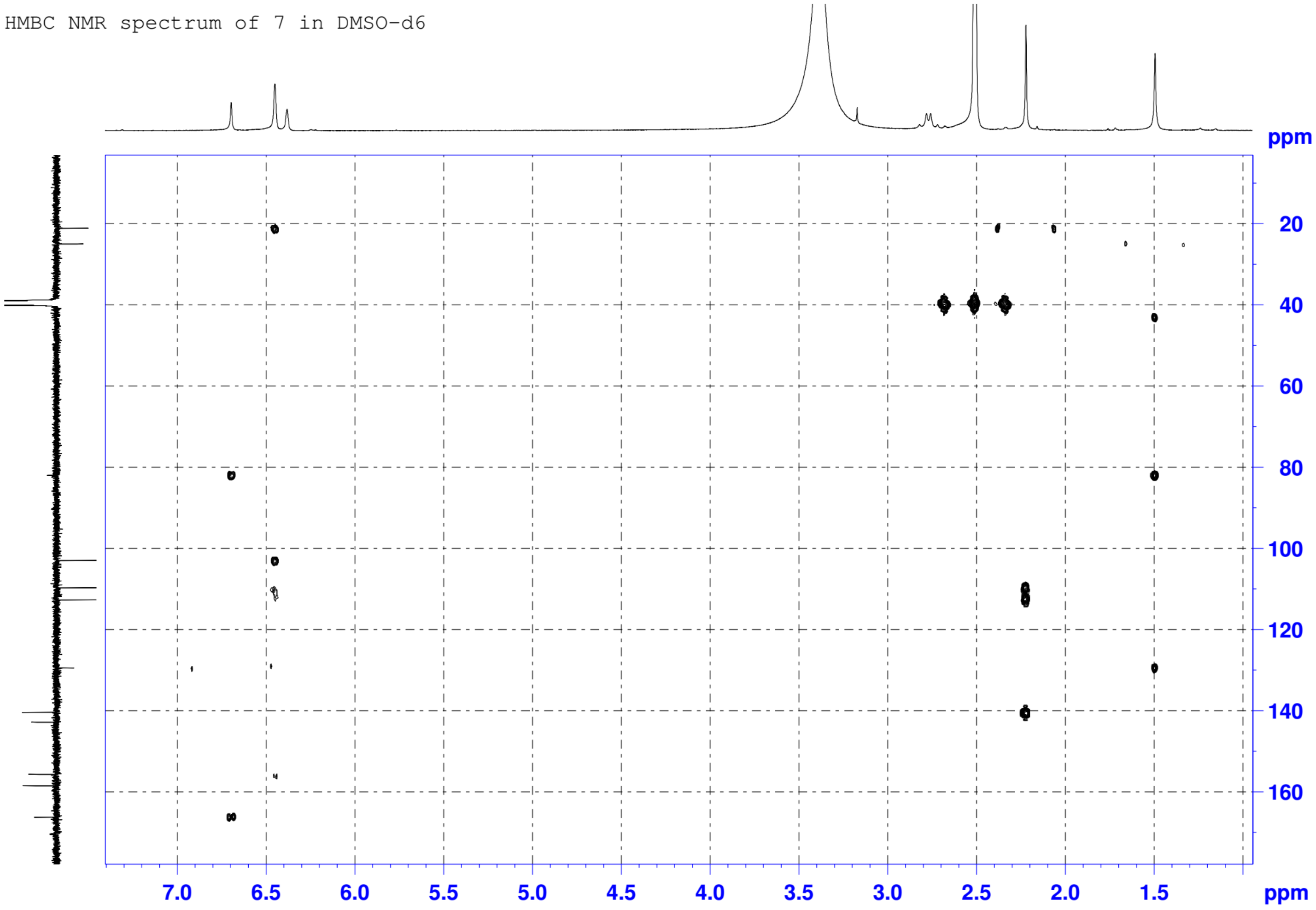

Figure S5-6. HMBC spectrum of **7** in DMSO-d_6.
